# Structural basis of telomeric nucleosome recognition by shelterin factor TRF1

**DOI:** 10.1126/sciadv.adi4148

**Published:** 2023-08-25

**Authors:** Hongmiao Hu, Anne-Marie M. van Roon, George E. Ghanim, Bilal Ahsan, Abraham O. Oluwole, Sew-Yeu Peak-Chew, Carol V. Robinson, Thi Hoang Duong Nguyen

**Affiliations:** ^1^ MRC Laboratory of Molecular Biology, Cambridge, CB2 0QH, UK.; ^2^ Department of Chemistry, University of Oxford, Oxford, OX1 3QZ UK.; ^3^ Kavli Institute for Nanoscience Discovery, University of Oxford, Oxford, OX1 3QU UK.

## Abstract

Shelterin and nucleosomes are the key players that organize mammalian chromosome ends into the protective telomere caps. However, how they interact with each other at telomeres remains unknown. We report cryo–electron microscopy structures of a human telomeric nucleosome both unbound and bound to the shelterin factor TRF1. Our structures reveal that TRF1 binds unwrapped nucleosomal DNA ends by engaging both the nucleosomal DNA and the histone octamer. Unexpectedly, TRF1 binding shifts the register of the nucleosomal DNA by 1 bp. We discovered that phosphorylation of the TRF1 C terminus and a noncanomical DNA binding surface on TRF1 are critical for its association with telomeric nucleosomes. These insights into shelterin-chromatin interactions have crucial implications for understanding telomeric chromatin organization and other roles of shelterin at telomeres including replication and transcription.

## Teaser

Shelterin factor TRF1 binds a telomeric nucleosome and induces a register shift in the nucleosomal DNA.

## INTRODUCTION

Mammalian telomeric DNA, composed of tandem telomeric TTAGGG repeats, is specifically bound by the shelterin complex (*1*, *2*). Shelterin maintains genome stability by protecting the chromosome ends from various DNA damage response and repair pathways (*1*). Telomere dysfunction and misregulation lead to tumorigenesis and various age-related diseases (*3*). Mammalian telomeres, like bulk genomic DNA, are also packaged into nucleosomes (*4*, *5*). Therefore, understanding how shelterin recognizes telomeric DNA in a chromatin environment is critical for understanding its protective role at telomeres. However, there is currently no available structural information on the interactions between shelterin and telomeric chromatin.

Shelterin consists of the six protein subunits: TRF1, TRF2, RAP1, TIN2, POT1, and TPP1. (*1*, *6*–*9*). Among these subunits, TRF1 and TRF2 each bind double-stranded (ds) telomeric DNA as preformed homodimers and thus associate shelterin with ds telomeric repeats. TRF1 and TRF2 are distant homologs and share two conserved domains: a TRF homology dimerization domain and a Myb oncoprotein-like (Myb) DNA binding domain (*8*, *10*). Crystal structures of TRF1 and TRF2 Myb domains with a telomeric dsDNA are nearly identical to each other (*11*). In each structure, two Myb domains of either TRF1 or TRF2 bind adjacent TAGGGTT motifs on the opposite faces of a dsDNA, mimicking the binding of the TRF1/TRF2 homodimer to telomeric DNA. The dsDNA in these structures adopted a linear conformation and was thus proposed to exclude nucleosome formation (*11*). Despite this observation, previous biochemical studies showed that TRF1 interacts with telomeric DNA in a nucleosomal context and that TRF1 alters nucleosome structure (*12*, *13*). In contrast, TRF2 DNA binding is strongly inhibited by telomeric nucleosomes, despite its similarity in DNA recognition to TRF1 (*14*). The mechanisms of how TRF1 interacts with and modulates telomeric nucleosomes were unknown. The molecular determinants contributing to the difference in nucleosome interaction of the two TRFs remained unclear.

Here, we determined the cryo–electron microscopy (cryo-EM) structures of an unbound human telomeric nucleosome core particle (teloNCP) and TRF1-bound teloNCP. The structures reveal a molecular basis of TRF1-nucleosome interaction. TRF1 binding to the nucleosome results in a register shift in the nucleosomal DNA. We define key residues and posttranslational modifications (PTMs) in TRF1 that confer its specificity for the teloNCP and yet are absent in TRF2 Myb domain, explaining the observed differences in TRF1 and TRF2 binding to the teloNCP.

## RESULTS

### Structure of a telomeric nucleosome reveals nucleosome positioning on telomeric DNA

**Fig. 1.**
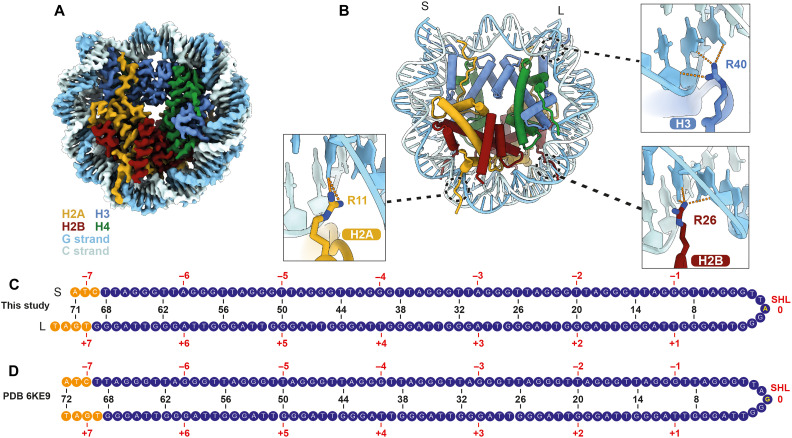
Structure of a human teloNCP. (**A**) Cryo-EM reconstruction (2.5 Å) and (**B**) the atomic model of a human teloNCP. Within the DNA duplex, the G-strand consists of the G-rich TTAGGG repeats. The C-strand consists of the CCCTAA repeats, which are complementary to the TTAGGG repeats. (**C**) DNA positioning of telomeric sequence observed in the structure shown in (A) and (B). Short (S) and long (L) labels denote the short and long lengths of the DNA on either side of the dyad, respectively. The numbers on top of the sequence (±1, ±2, …, ±7) denote the SHLs relative to the nucleosomal dyad position (SHL 0). The blue nucleotides represent the 23 telomeric repeats. The orange nucleotides represent the nontelomeric sequences resulting from restriction digestion of the DNA construct. The labels and coloring scheme in this figure is used throughout the manuscript. Base-specific interactions between DNA bases and histone H2A, H3, and H2A are shown in the three close-up views. (**D**) DNA positioning of telomeric sequence in the published teloNCP crystal structure (PDB 6KE9) (*16*).

The telomeric TTAGGG repeats were suggested to be unfavorable for DNA positioning on the nucleosome (*15*). To first define how the telomeric DNA sequence is positioned on the nucleosome, we reconstituted a human teloNCP with a 145-bp telomeric DNA containing 23 TTAGGG repeats and determined its cryo-EM structure to 2.5-Å resolution (Fig. 1A; figs. S1 and S2, A to D; tables S1 and S2; and data S1). In our structure, the dyad axis is positioned at an A-T base pair on the central telomeric repeat (Fig. 1, B and C, and fig. S2E). To further validate our DNA register assignment, we calculated the model-versus-map cross-correlation for models with each of the six possible options of DNA positioning on the telomeric DNA sequence (fig. S2F). The cross-correlation values of the assigned register are indeed the highest among the six possibilities (fig. S2F). The observed nucleosome positioning creates an asymmetry in the DNA lengths on either side of the dyad (Fig. 1, B and C). For clarity, we name the two ends of the nucleosomal DNA as short (S) and long (L) according to their lengths (Fig. 1, B and C).

A 2.2-Å crystal structure and 3.5-Å cryo-EM structure of teloNCP have been reported (*16*, *17*). However, there are three major differences between our structure and the published structures. First, the nucleosome dyad position of the published structures differs from that of our structure by one base (Fig. 1, C and D, and fig. S3, A to C). The electron density of the central telomeric repeat of the teloNCP crystal structure is ambiguous (fig. S3B), likely caused by phase errors arising from the presence of mixed nucleosome orientations in the crystal lattice (*16*). On the other hand, the 3.5-Å cryo-EM density was at insufficient resolution for accurate DNA sequence assignment (fig. S3C). Second, analyses of DNA helical parameters show that the DNA in our structure displays substantially less extreme geometries compared to the reported teloNCP structures (*16*) and more resembles that of the Widom 601-NCP structure (fig. S3D) (*18*). These discrepancies may be caused by the involvement of the DNA in crystal packing. Third, we resolved additional amino acids at the N termini of all four histone proteins. Notably, the basic tails of histone H2A, H2B, and H3 insert into the minor grooves near superhelical locations (SHLs) ±4, ±3, and ± 1, respectively, and form base-specific interactions via hydrogen bonding between three arginine residues [Arg^11^ (R11) of H2A, Arg^26^ (R26) of H2B, and Arg^40^ (R40) of H3] and DNA bases (Fig. 1B and fig. S2, G to I). These base-specific interactions are absent in most nucleosome structures, except two recent structures (*19*, *20*). As discussed below, the newly resolved extensions of the histones have a role in determining the binding site preference of the shelterin factor TRF1 on the teloNCP.

### TRF1-TIN2-TPP1 complex directly binds the teloNCP

**Fig. 2.**
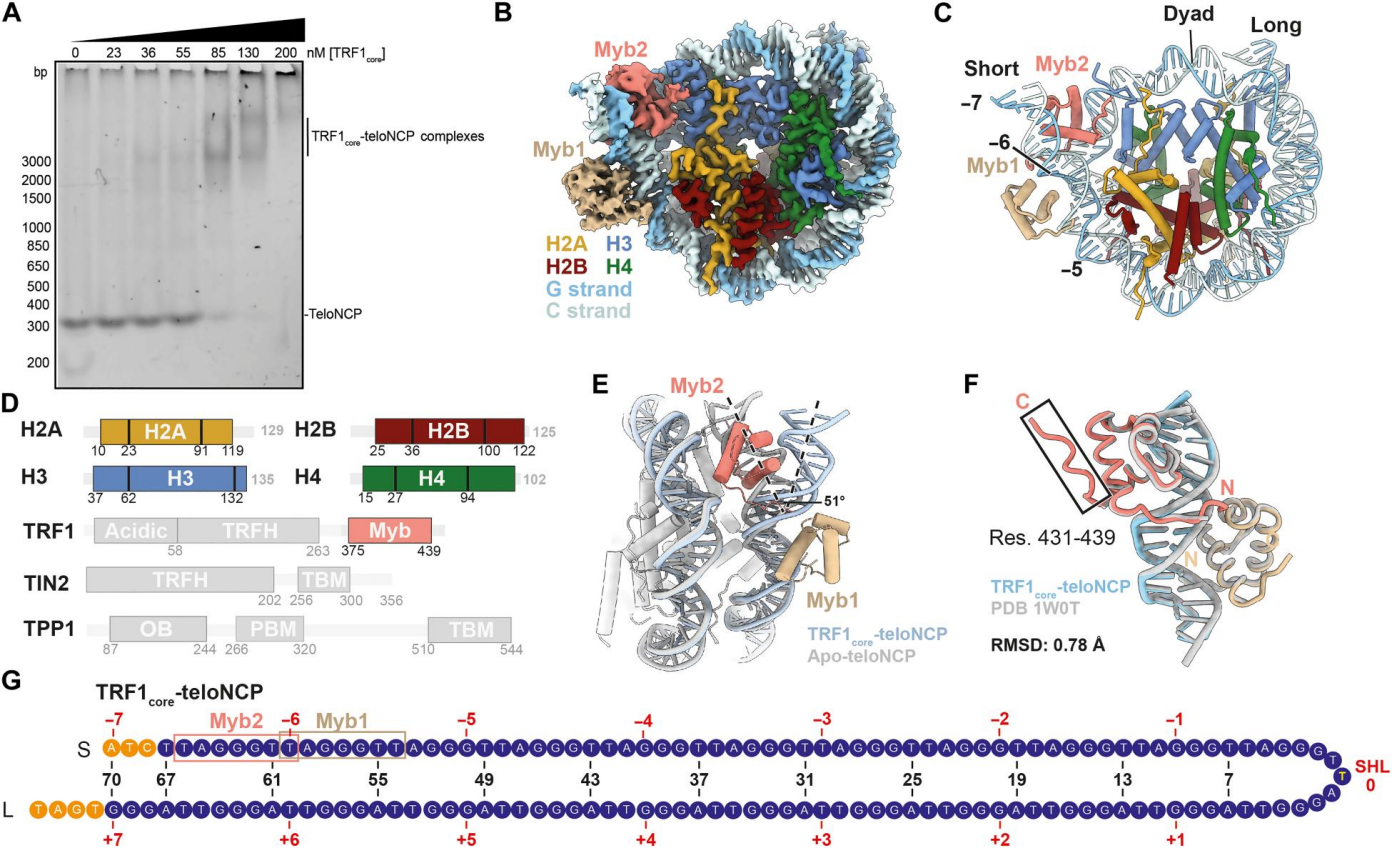
Structure of the 2:1 TRF1_core_-teloNCP complex. (**A**) EMSAs showing titration of TRF1_core_ against teloNCP. Experiments were performed in triplicate. (**B**) Cryo-EM reconstruction (2.7 Å) and (**C**) the atomic model of TRF1_core_-teloNCP complex, respectively. Subunits are colored as labeled. Dyad position and SHLs are indicated in (C). (**D**) Domain architectures of protein subunits in the complex. Unresolved regions are shown as semi-transparent. (**E**) Superimposition of the apo-teloNCP (gray) and TRF1_core_-teloNCP (colored) structures determined in this work to show unwrapping of nucleosomal DNA by TRF1. (**F**) Comparison of the structures of the Myb domains bound to naked telomeric DNA (PDB 1W0T, gray) (*11*) and bound to the nucleosomal DNA (colored). (**G**) Positioning of telomeric DNA sequence in the TRF1_core_-teloNCP structure. The TRF1 Myb domain binding sites are indicated.

Within shelterin, TRF1 forms stable interactions with TIN2, which, in turn, interacts with TPP1 (*21*, *22*). Thus, to mimic TRF1 in a more physiological context, we purified the shelterin TRF1-TIN2-TPP1 subcomplex (TRF1_core_). Native mass spectrometry (MS) indicates that this subcomplex exhibits a 2:2:2 stoichiometry (fig. S4, A and B). Electrophoretic mobility shift assays (EMSAs) show that the TRF1_core_ interacts with the teloNCP (Fig. 2A). We next isolated a complex of TRF1_core_-teloNCP by glycerol gradient centrifugation and confirmed complex formation by SDS–polyacrylamide gel electrophoresis (SDS-PAGE), native gel electrophoresis (fig. S4, C and D), and negative stain electron microscopy (EM) (fig. S5) before cryo-EM structure determination.

Purified TRF1_core_ alone runs as a single peak on the glycerol gradient (fig. S4E). Upon assembly with the teloNCP, we observed TPP1 and TIN2 being stripped from the TRF1_core_ complex in the top fractions of the gradient (fig. S4C), suggesting that binding to the teloNCP results in a change of the subunit stoichiometry of the TRF1_core_ complex. However, the low solubility of the TRF1_core_-teloNCP complex in a volative buffer required for native MS experiments precluded the determination of its molecular mass by native MS.

Cryo-EM analysis of the TRF1_core_-teloNCP complex revealed that a major population of particles contained two Myb domains of a TRF1 homodimer bound to the teloNCP (2:1 TRF1_core_-teloNCP complex) (Fig. 2, B and C; fig. S6, A to C; and tables S1 and S2). We resolved this structure to 2.7-Å overall resolution with a local resolution range of 2.7 to 4.0 Å (fig. S7, A to D). The resulting structure allows unambiguous modeling of the histone octamer, TRF1, and the telomeric DNA (Fig. 2, B and C; fig. S7, E to I; and data S2).

Although full-length TRF1, TIN2, and TPP1 are present in the cryo-EM sample (fig. S4C), only the two Myb domains of a TRF1 homodimer are resolved (Fig. 2, B to D). Despite the presence of weak density on top of the nucleosome in the two-dimensional (2D) class averages of the TRF1_core_-teloNCP complex (fig. S6B), 3D variability analysis (3DVA) revealed only the dynamics at the Myb domain–bound region of the nucleosome but not any additional density (fig. S8A and movie S1). Therefore, the unresolved parts of the complex are likely flexible, as suggested by another study (*23*). Furthermore, the TRF1 density bound to the nucleosome in the cryo-EM map is substantially smaller than the density observed in the negative-stain EM reconstruction of the complex (fig. S5D). This suggests partial denaturation of the complex during cryo-EM sample preparation possibly due to interactions with the air-water interface in addition to the inherent flexibility of the complex (*24*). Cryo–electron tomography (cryo-ET) experiments confirmed that most TRF1_core_-teloNCP particles partition to the air-water interfaces on the cryo-EM grids (fig. S8B).

### TRF1 binding induces a DNA register shift in the teloNCP

Our TRF1_core_-teloNCP structure reveals that 1.5 turns of double-helix DNA at the entry/exit site are unwrapped from the histone octamer, and TRF1 directly binds the unwrapped DNA. (Fig. 2, B and C). TRF1 binding changes the DNA trajectory by approximately 51° (Fig. 2, C and E). Consequently, the TRF1-bound region of the nucleosomal DNA adopts a linear conformation similar to that previously observed in the TRF1 Myb domain structure bound to naked telomeric DNA (Fig. 2F) (*11*). One Myb domain, termed Myb1, binds the outer DNA gyre spanning SHL −5.5 to −6.5, while the other Myb domain, termed Myb2, binds the adjacent inner DNA gyre by inserting between nucleosomal DNA and the histone octamer (Fig. 2, B, C, and E). The two modes of nucleosome binding by TRF1 are notably similar to that observed for various pioneering transcription factors, histone-modifying enzymes, and chromatin remodelers (fig. S9, A to F).

To understand the binding site preference, we examined all accessible Myb domain binding sites on the nucleosome (fig. S10). For all binding sites, except for the site between SHL ±5.5 and ± 6.5, steric clashes occur with the newly resolved histone tails (Fig. 1B), likely disfavoring Myb binding (fig. S10, A and B). Our results also rationalize a previous observation that TRF1 preferentially binds telomeric repeats at the end of the nucleosome rather than near the dyad axis (*12*).

Notably, the dyad position of the TRF1-bound nucleosome is shifted by one base compared to that of the apo-teloNCP structure (Figs. 1C and 2G and figs. S2E and S7E). Our DNA register assignment is also validated by cross-correlation calculations as described for the apo-teloNCP structure (fig. S7F). Consequently, the lengths of the DNA on either side of the dyad become more asymmetric (Fig. 2G). In support of our observations, previous nucleosome mobility and atomic force microscopy experiments demonstrated that TRF1 binding alters nucleosome positioning and spacing and also induces sliding of telomeric nucleosome (*12*–*14*). TRF1 is not known to have any adenosine triphosphatase or adenosine triphosphate (ATP) binding activity. Thus, our work provides the first structural evidence for an ATP-independent nucleosome modulation activity of shelterin.

### TRF1 binds both ends of the teloNCP

**Fig. 3.**
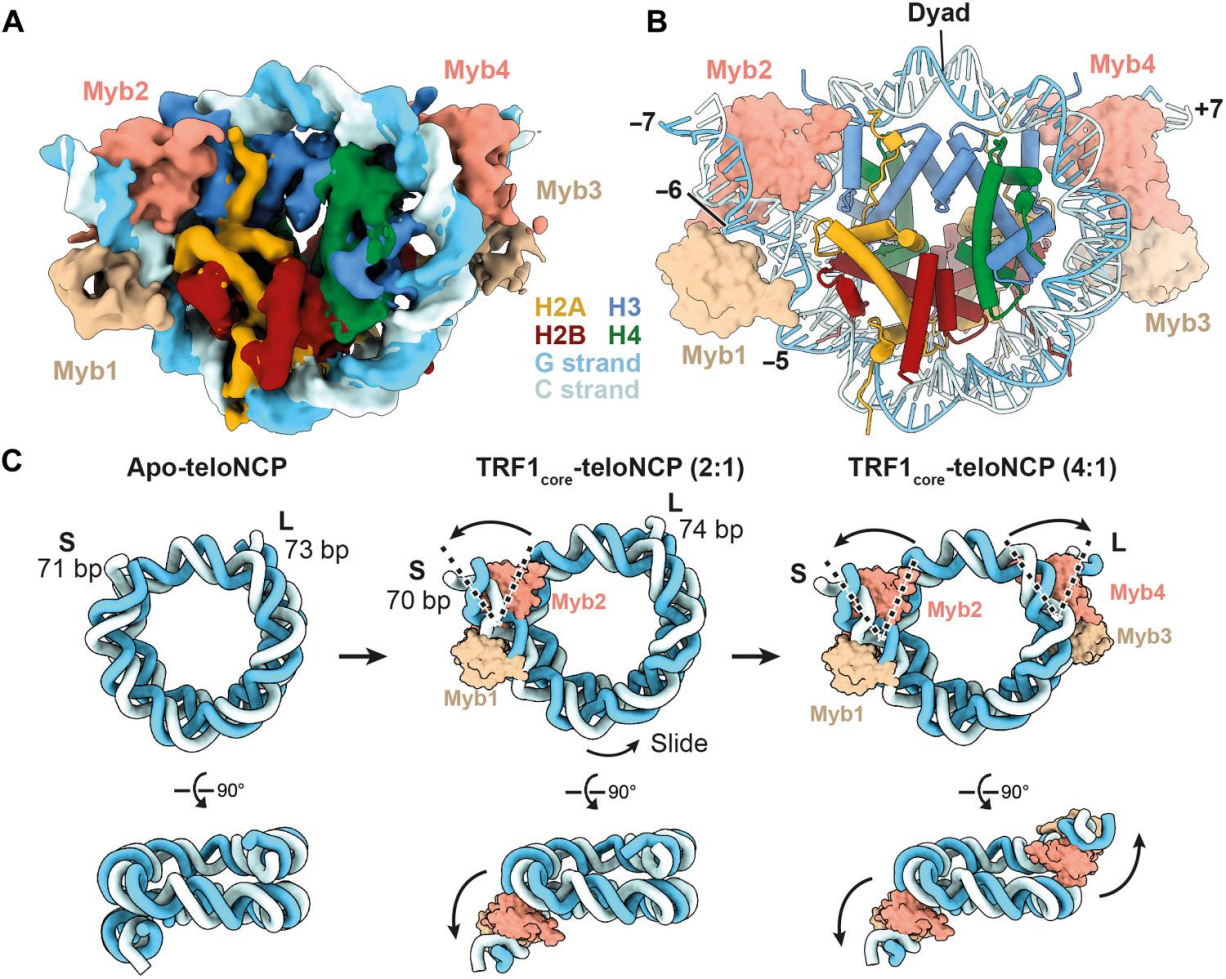
Structure of the 4:1 TRF1_core_-teloNCP complex. (**A**) Cryo-EM reconstruction (6.7 Å) and (**B**) the model of 4:1 TRF1_core_-teloNCP complex, respectively. Subunits are colored as labeled. Dyad position and SHL, which are occupied by the Myb domains of TRF1, are also indicated. (**C**) Model for the hierarchical assembly of TRF1_core_ on the teloNCP based on the structures of apo-teloNCP and the 2:1 and 4:1 TRF1_core_-teloNCP complexes determined in this study.

Through extensive 3D classification, we resolved a small subset of particles with four Myb domains bound to the nucleosome (4:1 TRF1_core_:teloNCP) to 6.7-Å resolution. This allowed rigid-body docking of the Myb domains and the nucleosome (Fig. 3, A and B; fig. S11, A to D; and data S3). In this structure, 1.5 turns of double-helix nucleosomal DNA at both the entry and exit sites are unwrapped with two Myb domains occupying each site, resulting in a more loosely packed nucleosome (Fig. 3, A to C). This is highly similar to the structure of a nucleosome bound to two copies of the nuclear receptor-binding SET domain 3 methyltransferase, which also engages the nucleosome via the same histone H3 region (fig. S9, G and H) (*25*). The ability of TRF1 to remodel two ends of the pseudosymmetric teloNCP further reinforces its similarity to other chromatin remodeling/modifying complexes.

EMSA data showed two-step binding of TRF1_core_ to the teloNCP with increasing TRF1_core_ concentrations (Fig. 2A), suggesting a step-wise formation of the 4:1 complex. Therefore, we propose that two Myb domains first bind one end of the nucleosome and slide the DNA toward the other end to form the 2:1 complex, and then a second pair of Myb domains remodels the other end to form the 4:1 complex (Fig. 3C). Under our purification conditions, native gel analysis showed that the majority of the TRF_core_-teloNCP complex were 2:1 (fig. S4D), rationalizing the relative particle distributions of the 2:1 and 4:1 complexes observed in the cryo-EM data.

### Phosphorylation of TRF1 facilitates binding to histone octamer

**Fig. 4.**
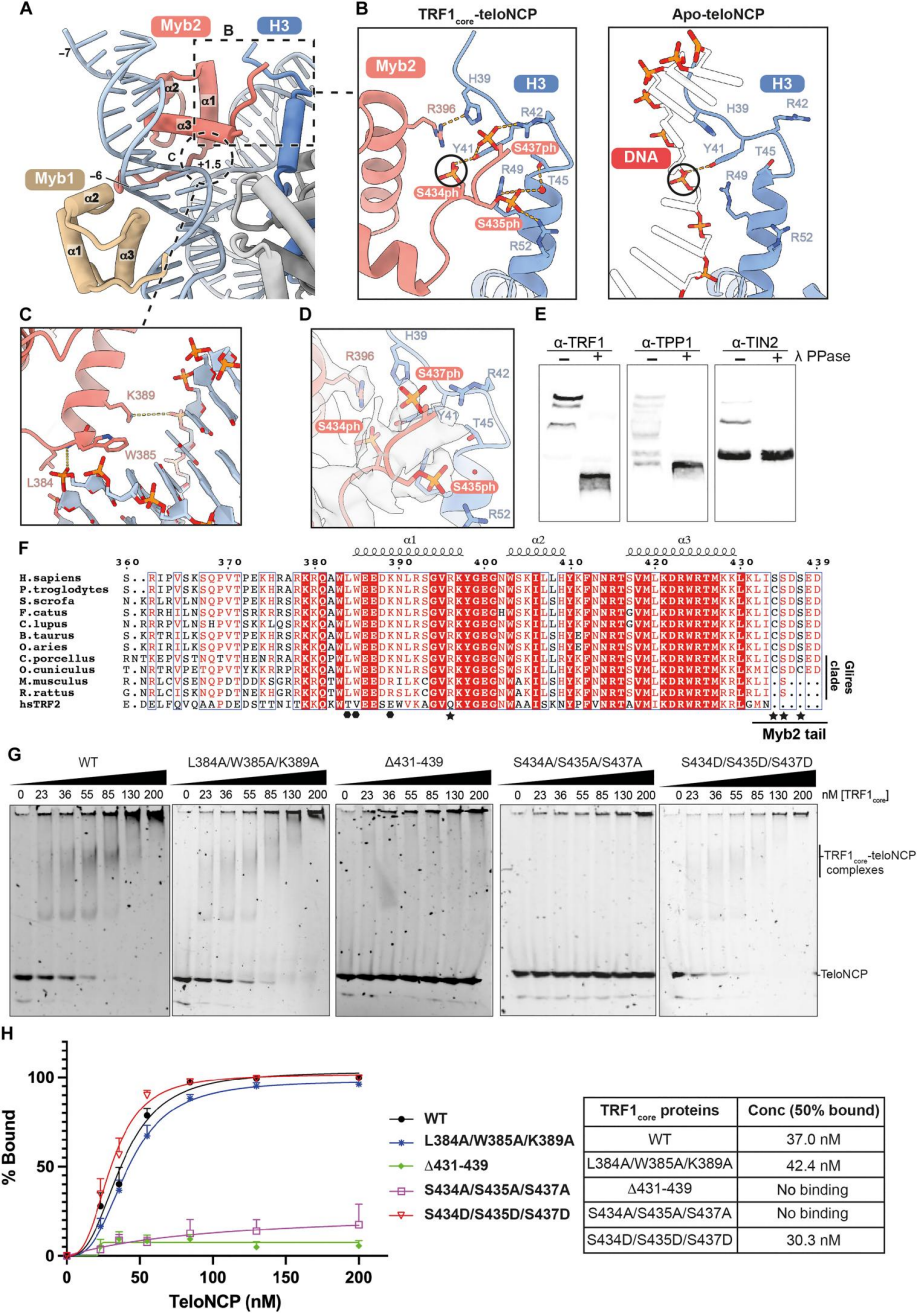
Noncanonical DNA interaction surface and phosphorylation of TRF1 crucial for nucleosome binding. (**A**) Interactions between the two TRF1 Myb domains with teloNCP. Dashed box and circle indicate histone H3-Myb2 and DNA-Myb2 interactions, respectively. (**B**) Left: Close-up view of the interaction between the phosphorylated C-terminal residues of TRF1 Myb2 domain with histone H3 N-terminal tail in the TRF1_core_-teloNCP structure. Right: Close-up view of the same region of histone H3 interacting with DNA in the apo-teloNCP structure. Black circles highlight the observation that the phosphate group of S434 occupies the same position as a DNA backbone phosphate. (**C**) Close-up view showing noncanonical DNA interactions made by helix 1 of Myb2. (**D**) Cryo-EM density of the phosphorylated C-terminal residues of TRF1 Myb2 domain. (**E**) Phos-tag gels of the untreated and λ-phosphatase (λ PPase) treated TRF1_core_ samples combined with immunoblotting using TRF1, TPP1, and TIN2 antibodies (α-TRF1, α-TPP1, and α-TIN2). (**F**) Sequence alignment of TRF1 Myb domains from various mammalian species and human TRF2 Myb domain. The hexagonal dots and stars underneath the sequence denote residues involved in DNA interaction within the noncanonical DNA surface on Myb2 (C) and residues involved in interactions with histone H3 (B), respectively. (**G**) EMSAs showing titration of purified wild-type (WT) and mutant TRF1_core_ complexes against teloNCP. Experiments were performed in triplicate. (**H**) Quantification of EMSA experiments shown in (G). In the left, we plotted percentages of unbound teloNCPs as a function of protein concentration in the EMSA reaction of the wild-type and each mutant complex. The right table shows the concentration of each TRF1_core_ complex at which 50% of teloNCP remains unbound as determined from the graphs. Error bars at each concentration point are the SEM obtained from the three replicates.

Our structure reveals the first direct interactions between shelterin and histones. We resolved the acidic C terminus of Myb2 (residues 431 to 439), which was disordered in the previous crystal structure of TRF1 Myb domain (Figs. 2F and 4, A and C) (*11*). The Myb2 tail extensively interacts with the basic N-terminal tail of histone H3 (residues 39 to 53), displacing the DNA phosphate backbone found in the same region in the apo-teloNCP structure (Fig. 4B, right). Truncating the C terminus of TRF1 (residues 431 to 439) (Δ431-439) abolished the binding of TRF1_core_ to teloNCP (Fig. 4, G and H, and fig. S12A). Consistent with our observation, previous study demonstrated that trypsin treatment of the nucleosome to remove histone tails reduced TRF1 binding to the nucleosome (*14*). Our data suggest that the interactions between the C terminus of TRF1 and histone H3 are critical for TRF1_core_ binding to the teloNCP.

We observed density consistent with phosphorylated Ser^434^ (S434), Ser^435^ (S435), and Ser^437^ (S437) at the C terminus of TRF1 (Fig. 4D and fig. S7, H to J). The phosphoserine side chain of S434 hydrogen bonds with Tyr^41^ (Y41) of histone H3, mimicking the DNA backbone phosphate observed in the apo-teloNCP structure (Fig. 4B). The phosphoserine side chains of S435 and S437 are located in a positively charged pocket on histone H3 and are coordinated by numerous histone H3 residues and water molecules (Fig. 4B, left). Lambda phosphatase treatment combined with Phos-tag PAGE showed that all three components of the TRF1_core_ complex (TRF1, TIN2, and TPP1) were phosphorylated in our sample, likely by endogenous insect cell kinases (Fig. 4E). Phosphorylation of S434, S435, and S437 has been previously reported in various proteomic studies in both human and mouse cells (fig. S12F) (*26*–*31*). Native MS of the TRF1_core_ complex yielded a higher molecular weight than predicted, suggesting the presence of PTMs (fig. S4B). Further tandem MS (MS/MS) analyses confirmed that S434, S435, and S437 are among the phosphorylated residues in our protein sample (fig. S12, D and E).

To further understand the role of the phosphoserines in nucleosome binding, we prepared TRF1_core_ with the TRF1 S434A/S435A/S437A triple mutation, which largely abolished teloNCP binding (Fig. 4, G and H, and fig. S12A). On the other hand, TRF1 phospho-mimetic mutant S434D/S435D/S437D binds the teloNCP with a higher affinity than the wild-type TRF1_core_ (Fig. 4, G and H, and fig. S12A). While the kinase(s) responsible for phosphorylation of S434 and S437 have not been identified, S435 is phosphorylated by Polo-like kinase 1 (Plk1) (*32*). Consistent with our data, previous work showed that phosphorylation of S435 by Plk1 markedly increased TRF1 binding to telomeres in vitro and in vivo (*32*). In our MS data, S435 is phosphorylated in 87.3% of the identified peptides, explaining why additional treatment of our TRF1_core_ sample with Plk1 only slightly increased its affinity to the teloNCP (fig. S12, B and C). Together, our data suggest that TRF1 association with telomeres depends on not only specific interactions with telomeric DNA but also interactions between the phosphorylated C terminus of TRF1 and the histone octamer.

### Conserved molecular features crucial for the binding of TRF1 to the teloNCP are absent in TRF2

Residues involved in the interactions between the C terminus of TRF1 and histone H3 are highly conserved in mammals (Fig. 4F and fig. S13B). S435 is conserved in all mammalian TRF1, whereas rodent TRF1 lacks S434 and S437 (Fig. 4F). All mammalian TRF1 proteins contain one or more of the three serine residues. Because of the inability of the TRF1 S434A/S435A/S437A and Δ431-439 mutants to bind the teloNCP, we propose that mammalian TRF1 uses at least one of these phosphoserines for interaction with telomeric chromatin. Furthermore, the C-terminal region of TRF1 encompassing the three phosphoserines is absent in TRF2 (Fig. 4F) and thus would likely contribute to the difference in nucleosome recognition between TRF1 and TRF2.

Our structure also revealed a noncanonical DNA binding site on TRF1. Each TRF1 Myb domain consist of three α helices: helices 1, 2, and 3 (Fig. 4A). For both Myb1 and Myb2, we observe canonical interactions with the telomeric TAGGGTT motifs made by helices 2 and 3 and the N-terminal loop preceding helix 1 (*11*). In our structure, helix 1 (residues 384 to 396) of Myb2 makes a secondary contact with the DNA major groove at SHL +1.5 (Fig. 4A). Leu^384^ (L384), Trp^385^ (W385), and Lys^389^ (K389) of helix 1 form numerous interactions with the DNA (Fig. 4C and fig. S7G). The discovered DNA binding site on helix 1 is solvent exposed in Myb1 (Fig. 4A) and in the previous structure of TRF1 Myb domain [Protein Data Bank (PDB) 1W0T] (*11*). Although our biochemical data showed that the C terminus of TRF1 is essential for nucleosome binding, we also prepared a TRF1_core_ L384A/ W385A/K389A triple mutant to test its contribution to teloNCP binding. This mutant showed a slightly decreased affinity to the teloNCP compared to the wild-type complex (Fig. 4, G and H, and fig. S12A). Therefore, our results suggest that helix 1 of TRF1 Myb domain also contributes to nucleosomal DNA binding.

L384, W385, and K389 are conserved among all mammalian TRF1 homologs but not in TRF2 (Fig. 4F), suggesting a contribution of this surface to the higher affinity of TRF1 for the nucleosome than TRF2 (*14*). Substitution of residues L371 and W372 in mouse TRF1 (equivalent to human L384 and W385, respectively) for the corresponding residues in TRF2 resulted in a fragile telomere phenotype and telomere replication defects in vivo (*33*). The observed defects were linked to the inability of the mutant TRF1 to recruit transcription factor II H (TFIIH) to promote telomere replication. However, it remains unknown whether L384 and W385 directly bind TFIIH and whether DNA binding at these residues influences TFIIH recruitment. On the basis of the observed involvement of these residues in remodeling the nucleosome, we propose that this second DNA binding surface on TRF1 can also promote telomere replication fork progression by remodeling telomeric chromatin, contributing to the defects observed in vivo.

## DISCUSSION

Our data indicate that TRF1 exhibits the functional features of pioneer transcription factors and chromatin remodelers, which would explain its various noncanonical roles besides telomere protection. TRF1 is known to promote telomere replication by recruiting the BLM helicase to resolve telomeric G-quadruplexes during replication (*34*, *35*). However, TRF1-deficient cells exhibit more severe telomere replication defects than BLM-deficient cells (*34*, *35*). Thus, we propose that TRF1 unwrapping of telomeric nucleosomes provides an additional mechanism by which TRF1 facilitates replication.

TRF1 plays critical roles in transcriptional programming of pluripotent stem cells by regulating the recruitment of polycomb repressive complex 2 to key pluripotency and differentiation genes in mouse (*36*, *37*). Chromatin immunoprecipitation sequencing data found that TRF1 binds not only a set of genes, which contain TTAGGG repeats and are also targets of the pluripotency regulator ZFP322A, but also extratelomeric sites (*37*, *38*). Plant telomere-repeat binding factors homologous to TRF1 are known transcription factors, which recruit PRC complexes to promoters containing specific telobox motifs (*39*). On the basis of the remarkable similarity in nucleosome binding between TRF1 and the pluripotency pioneer transcription factors, OCT4 and SOX2 (fig. S9, B and C) (*40*), we speculate that TRF1 may act as a transcription factor in stem cells.

**Fig. 5.**
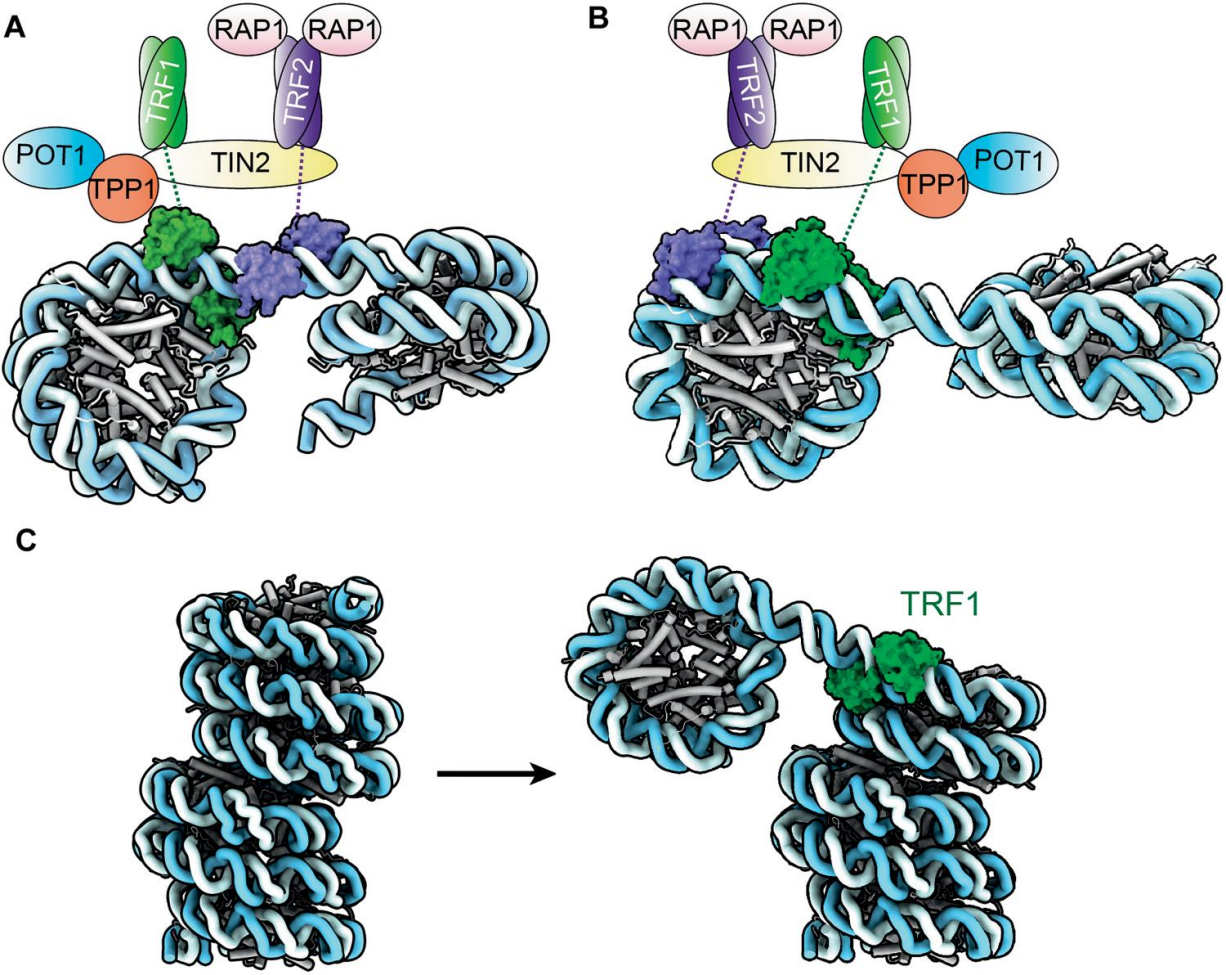
Model for the organization of shelterin and nucleosomes on telomeric DNA. (**A** and **B**) Two proposed models for how shelterin and telomeric nucleosomes are organized on telomeric DNA. In both models, TRF1 binds at the junction between nucleosome and linker DNA as shown in our structures. TRF2 binds either the linker DNA (in A) or on the outer DNA gyres (in B). The nucleosome repeat length in these models is 157 bp, based on the published work (*5*). (**C**) Remodeling of the columnar telomeric chromatin structure (PDB 7V9K) (*17*) to accommodate TRF1 binding.

Our structures of the TRF1-bound teloNCP have important implications for our understanding of how shelterin and nucleosomes both occupy telomeres. Previous studies proposed that shelterin binds the linker DNA between nucleosomes (*11*) or spanning adjacent outer nucleosomal DNA gyres (*17*). However, in this work, we resolved a stable binding mode of TRF1 at the junction between the nucleosome and linker DNA. TRF2 could bind at either the linker DNA (Fig. 5A) or nearby outer nucleosomal DNA gyres (Fig. 5B). This binding mode of shelterin to telomeric chromatin would disfavor the linker histone H1 binding at the entry/exit sites, rationalizing the underrepresentation of histone H1 at telomeres (fig. S13G) (*41*). We also cannot exclude the possibility that TRF1 binds additional sites when provided with a teloNCP with a longer DNA linker or a nucleosome array. Therefore, future work will be needed to elucidate additional shelterin-chromatin interactions.

Recent work showed that under low salt conditions, in vitro reconstituted telomeric chromatin fibers form a columnar structure of closely stacked nucleosomes without linker DNA in between (Fig. 5C) (*17*). To accommodate the TRF1 binding as observed in our structures, the nucleosome stacking in the columnar structure would need to be disrupted. Furthermore, the reported chromatin fiber structure has a nucleosome repeat length of 132 bp, which is 18 to 33 bp shorter than that determined by nuclease digestion of human and rat telomeric chromatin (*4*, *5*). Thus, binding of shelterin would likely remodel these chromatin fibers to create longer nucleosome spacing as observed in vivo (Fig. 5C). Both TRF1 and TRF2 have been shown to alter nucleosome spacing in vitro (*14*). Besides the phosphorylation of TRF1 observed in this study, we found that TRF1 Myb2 directly interacts or is close proximity to residues of histone H3 and H2A known to have PTMs (fig. S13, A to F) (*42*). Therefore, PTMs of both shelterin and histones could additionally regulate shelterin-chromatin interactions, allowing dynamics in telomere structures through different stages of the cell cycle.

## MATERIALS AND METHODS

### Human histone octamer purification

Human histone H2A/H2B dimer was purified as described previously (*43*). Plasmid pRSFDuet-H3-H4 (a gift from K. Muir and D. Barford, Medical Research Council, Laboratory of Molecular Biology) with an N-terminal His_6_-HRV-3C site fusion on H4 was transformed into Rosetta(DE3)-pLysS cells. HRV-3C is human rhinovirus 3C protease cleavage site. Twelve liters of transformed cells was cultured at 37°C in 2xTY media supplemented with kanamycin (50 μg/ml) and chloramphenicol (35 μl/ml) to optical density at 600 nm = 0.4. The temperature was then lowered to 18°C for 1 hour before induction with 0.5 mM isopropyl-β-d-thiogalactopyranoside followed by an overnight incubation. Harvested cells were resuspended in nickel–nitrilotriacetic acid (Ni-NTA) buffer A [50 mM tris-HCl (pH 8.0), 2 M NaCl, 30 mM imidazole, 1 mM phenylmethylsulfonyl fluoride (PMSF), and 5 mM β-mercaptoethanol], containing 0.01% Igepal CA-630 (MP Biomedicals, catalog no. 198596, lot 5917 J) and cOmplete protease inhibitor cocktail (Roche) and lysed by sonication. The lysate was cleared by centrifugation at ~48,000*g* for 20 min at 4°C. The resulting supernatant was loaded on a 5-ml HisTrap HP column (Cytiva) equilibrated with Ni-NTA buffer A. The proteins were eluted by a step elution to 100 mM imidazole followed by a 100 to 500 mM linear gradient of imidazole. Peak fractions were pooled and five times diluted with 50 mM tris-HCl (pH 8.0), 1 mM PMSF, and 1 mM dithiothreitol (DTT), loaded onto a 5-ml HiTrap SP HP column (Cytiva) equilibrated in IEX buffer A [50 mM tris-HCl (pH 8.0), 500 mM NaCl, 1 mM PMSF, and 1 mM DTT] and eluted with a 0.5 to 2 M linear gradient of NaCl. Peak fractions were pooled and NaCl was added up to 2 M final concentration, and histone dimer H3/His-H4 was concentrated using an Amicon-Ultra-15 concentrator (Millipore) with an exclusion size of 10 kDa. The protein complex was flash-frozen and stored at −70°C until further use.

H3/His-H4 and His-H2A/His-H2B was mixed at 1:1.2 ratio. Fifty microliters of PreScission protease (5.2 mg/ml, a gift from the Passmore lab) per 8 mg total histones was added, and the sample was incubated in the cold room for 2 hours. Completion of cleavage was checked on a 4 to 12% bis-tris SDS-PAGE gel (Thermo Fisher Scientific). After complete cleavage, 500 μl of glutathione Sepharose 4B beads (Cytiva) was added to the sample and incubated for 30 min on ice. The resin was removed over a filter. The untagged octamer was concentrated using an Amicon-Ultra-15 concentrator (Millipore) with a molecular weight cutoff of 10 kDa and loaded onto a HiLoad 16/600 Superdex 200 column (Cytiva), pre-equilibrated in SEC buffer [50 mM tris-HCl (pH 8.0), 2 M NaCl, 1 mM EDTA, and 1 mM DTT]. Peak fractions were pooled, concentrated, snap-frozen in liquid nitrogen, and stored at −70°C until further use.

### TeloNCP reconstitution

The plasmid containing eight copies of 145-bp telomeric DNA [5′-ATC-(GGGTTA)_23_-TGAT-3′] repeats flanked by Eco RV was a gift from the Nordenskiöld’s lab. DNA preparation and teloNCP reconstitution with human histone octamer were carried out as previously published (*16*). Reconstituted teloNCP was loaded onto a Superose 6 increase 10/300 GL column (Cytiva), pre-equilibrated in 20 mM tris (pH 7.4), 150 mM KCl, 1 mM EDTA, and 1 mM DTT. Peak fractions were pooled, concentrated, and stored at 4°C until further use.

### TRF1_core_ complex purification

TRF1_core_ was expressed in insect cells following a similar procedure previously described in (*44*). Open reading frames of residues 87 to 544 of TPP1 (UniProtKB: A0A590TQL1), TRF1 (UniProtKB: P54274), and His8-MBP-SUMO*-TIN2 (UniProtKB: Q9BSI4) were cloned into a single expression vector by the biGBac method (*45*). Recombinant baculoviruses were generated by Bac-to-Bac Baculovirus Expression System (Invitrogen) using EmBacY cells (Geneva Biotech). One liter of *Spodoptera frugiperda* (Sf9) (Sf9, Oxford Expression Technologies Ltd., catalog no. 600100) cells at a density of 1.0 × 10^6^ cells/ml were infected with 10 ml of a high titer baculovirus stock. Infected cells were grown for 72 hours at 27°C. After harvesting by centrifugation, cell pellets were washed with phosphate-buffered saline (PBS) and resuspended in lysis buffer [25 mM Hepes-NaOH (pH 8.0), 300 mM NaCl, 1 mM MgCl_2_, 10 μM ZnCl_2_, 1 mM PMSF, 1 mM DTT, and 4× cOmplete protease inhibitor tablets (Roche, catalog no. 11873580001)]. Cell suspension was sonicated and clarified by centrifugation in two steps: first at 25,000*g* for 30 min and then ~142,000*g* for 45 min. The resulting supernant was filtered through a 0.22-μm filter before application to 25 ml of pre-equilibrated dextrin Sepharose resins (Cytiva, catalog no. 28-9355-97). The resin was washed three times with 10 column volumes (CVs) of wash buffer [25 mM Hepes-NaOH (pH 8.0), 300 mM NaCl, 1 mM MgCl_2_, 10 μM ZnCl_2_, 1 mM PMSF, and 1 mM DTT] and once with one CV wash buffer supplemented to 5 mM ATP and 4 mM MgCl_2_. Complexes were eluted from the resin by overnight incubation with SUMOstar protease (LifeSensors, catalog no. SP4110) at 4°C. NaCl concentration was adjusted to 150 mM by dilution before application to a 5-ml HiTrap heparin HP column (Cytiva, catalog no. 17040701), pre-equilibrated in heparin buffer [25 mM HEPES-NaOH (pH 8.0), 150 mM NaCl, 1 mM MgCl_2_, 10 μM ZnCl_2_, 0.01% IGEPAL CA-630, 1 mM PMSF, and 1 mM DTT]. Complex was eluted over a linear gradient of 150 mM NaCl to 1000 mM NaCl. Peak fractions were analyzed by SDS-PAGE, pooled, concentrated to 20 μM, dialyzed overnight into storage buffer [25 mM Hepes-NaOH (pH 8.0), 150 mM NaCl, 1 mM MgCl_2_, 10 μM ZnCl_2_, 0.01% IGEPAL CA-630, 10% glycerol, 1 mM PMSF, and 1 mM DTT], snap-frozen in liquid nitrogen, and stored at −70°C until use.

### Preparation of TRF1_core_ mutants

Mutagenesis primers were designed with NEBaseChanger. pACEBac1vector with TRF1 mutants was prepared with the NEB Q5 Site-Directed Mutagenesis Kit (New England Biolabs, catalog no. E0554). Constructs were transformed to chemically competent cells and grown at 37°C overnight. The presence of the mutations was confirmed by DNA sequencing. Recombinant baculoviruses were generated by Bac-to-Bac Baculovirus Expression System (Invitrogen) using EmBacY cells (Geneva Biotech). For the expression of wild-type and mutant MBP-tagged TRF1_core_ proteins, three individual viruses that contains TRF1, TIN2, and TPP1, respectively, were used together to infect Sf9 cells. Purification of MBP-tagged TRF1_core_ proteins followed the similar procedure as untagged protein purification. Proteins were first purified using dextrin Sepharose resin and eluted using wash buffer containing 50 mM maltose. Proteins were then diluted and further purified using a 1-ml HiTrap heparin HP column (Cytiva, catalog no. 17040601). Peak fractions from linear elution gradient were pooled, concentrated, snap-frozen in liquid nitrogen, and stored at −70°C until use.

### Electrophoretic mobility shift assays

TeloNCP (20 nM) was mixed with increasing amounts of either wild-type or mutant TRF1_core_ (0 to 200 nM) in EMSA binding buffer [20 mM Hepes-KOH (pH 7.5), 150 mM KCl, 1 mM MgCl_2_, 1 mM DTT, 0.01% Igepal, bovine serum albumin (0.5 mg/ml), and 5% glycerol] in 20 μl of final reaction volume. Binding reactions were incubated on ice for 30 min. TRF1_core_-NCP complexes were resolved on a 4% polyacrylamide (37.5:1 acrylamide:bis-acrylamide) native gel run in 0.25× tris-borate EDTA at 2 W for 30 min at room temperature. The gels were stained with CYBR Safe (Invitrogen, catalog no. S33102) for 30 min before being imaged on a Gel Doc system (Bio-Rad). All experiments were performed three times or more and yielded similar results.

For EMSA with Plk1-treated TRF1_core_, 1 μM TRF1_core_ and 0.5 μM Plk1 were incubated in a 20-μl final volume in a buffer containing 20 mM tris-HCl (pH 7.4), 150 mM KCl, 1 mM EDTA, 0.5 mM ATP, and 1 mM DTT at 30°C for 30 min. The Plk1-treated TRF1_core_ was diluted to a final TRF1_core_ concentration ranging between 0 and 200 nM for the EMSA. Binding reactions were performed as described above. All experiments were performed three times and yielded similar results.

Unbound nucleosome bands EMSA were quantified using ImageQuant (Cytiva). The percentage of bound nucleosome was calculated by (1 − *s*/*s*_0_)*100% (*s*, quantified signal; *s*_0_, the teloNCP signal when no TRF1_core_ is added). At each concentration, we averaged the percentages of bound nucleosome from three independent replicates and obtained the error bar. Nonlinear regression fitting was performed using GraphPad Prism 9 using the method: “Specific binding with Hill slope.” Concentrations of the teloNCP at which 50% of TRF1_core_ is bound was calculated and compared.

### Native MS

Purified protein at 20 μM was thawed, dialyzed into 750 mM ammonium acetate (pH 8.0) and 1 mM DTT overnight at 4°C. The next day, the sample was spun at 21,000*g* at 4°C to remove any precipitate, aliquoted, snap-frozen in liquid nitrogen, and stored at −70°C until use. Three microliters of the protein sample was transferred into a gold-coated borosilicate capillary (Harvard Apparatus) prepared in-house, which was then mounted on the nano-ESI source of a Q-Exactive hybrid quadrupole-Orbitrap UHMR mass spectrometer (Thermo Fisher Scientific, Bremen, Germany). Spectra were acquired using the capillary voltage of 1.2 kV, S-lens radio frequency of 200%, argon ultra-high vacuum pressure of 3.3 × 10^−10^ mbar, capillary temperature of 200°C, and resolution of 8750. Protein ions were activated using in-source trapping voltage of −100 V and an HCD voltage of 200 V. The noise level was set at 3, and voltages of the ion transfer optics injection flatapole, interflatapole lens, bent flatapole, and transfer multipole were set to 5, 3, 2, and 30 V, respectively. Data were visualized and exported for processing using the Qual browser of Xcalibur 4.1.31.9 (Thermo Fisher Scientific), and spectral deconvolution was performed using UniDec software (*46*). All measurements were performed at least three times and yielded similar results.

### Phos-tag PAGE

Forty micrograms of purified protein was diluted into 20 μl of storage buffer supplemented to 500 mM NaCl and 1 mM MnCl_2_ and then dephosphorylated with 200 U of lambda protein phosphatase (New England Biolabs, P0753L) for 4 hours at room temperature. Samples were diluted 1:40 in laemmli buffer and then resolved on a 100 μM Zn^2+^ 10% Phos-tag gel (AlphaLabs, AAL-107S1). After electrophoresis, the gel was first washed in transfer buffer [10 mM CAPS (pH 11.0) and 10% methanol] supplemented to 1 mM EDTA for 10 min at room temperature, washed in transfer buffer for 10 min at room temperature, and then transferred onto a nitrocellulose membrane. The membrane was blocked with 5% nonfat milk in PBS supplemented with 0.2% Tween 20 (PBST) for 1 hour, washed with PBST, and then incubated with primary antibodies overnight at 4°C. The membrane was then washed with PBST, incubated with secondary antibodies (Abcam) in PBST for 1 hour at 4°C, and then washed again with PBST before being imaged on a LI-COR Odyssey imager. The primary antibodies used were rabbit anti-TRF1 (1:1000; Proteintech, catalog no. 11899-1-AP, lot no. 00007098), rabbit anti-TPP1 (1:2000; Proteintech, catalog no. 25849-1-AP, lot no. 00025407), and rabbit anti-TIN2 (1:1000; Proteintech, catalog no. 11368-1-AP, lot no. 00043568). The secondary antibodies used were goat anti-rabbit Alexa Fluor 790 (1:5000; Abcam, catalog no. ab175781, lot no. GR226409-8).

### Identification of phosphopeptides by MS/MS

Purified protein was first subject to disulfide reduction with 5 mM TCEP for 15 min at room temperature and then alkylated with 10 mM iodoacetamide for 30 min at room temperature. Excess iodoacetamide was quenched with 10 mM DTT for 15 min at room temperature followed by methanol-chloroform precipitation. The pellet was resuspended with 80 μl of 20 mM Hepes NaOH (pH 8.0) and 8 M urea, aliquoted, then snap-frozen in liquid nitrogen, and stored at −70°C until use.

Protein sample in 8 M urea and 20 mM Hepes NaOH (pH 8) was reduced with 5 mM DTT, alkylated with 10 mM iodoacetamide, and digested with Lys_N (Promega) overnight at 30°C. Digested peptide mixture was desalted using home-made C18 stage tips (3M Empore) filled with poros R3 (Thermo Fisher Scientific) resin. Bound peptides were eluted with 30 to 80% acetonitrile (MeCN) /0.5% formic acid (FA) and partially dried down in a SpeedVac (Savant).

Iron-coated PHOS-Select metal chelate beads (Sigma-Aldrich) were washed five times with 30% MeCN/0.25 M acetic acid (loading buffer) and made into 50% slurry. To enrich for phosphopeptides, sample was resuspended in 100 μl of loading buffer, and 20 μl of PHOS-Select beads was added. Beads were shaken at room temperature for 45 min and then transferred to C8 stage tip (3M Empore). Beads in stage tip were washed four times with loading buffer, and phosphopeptides were eluted twice with 0.4 M ammonia solution, followed by once with 50%MeCN/0.5% FA. Eluates were acidified with FA, SpeedVac to remove MeCN, and desalted using home-made C18 (3M Empore) stage tip, same as above.

Liquid chromatography (LC)–MS/MS data acquisition was carried out on a Q Exactive HF-X mass spectrometer (Thermo Fisher Scientific) equipped with an Ultimate 3000 RSLC nano System (Thermo Fisher Scientific). Peptides were separated on an Easy-spray Pepmap C18 column, using buffer A [5% dimethyl sulfoxide (DMSO), 95% water, and 0.1% FA] and buffer B (5% DMSO, 75% MeCN, 20% water, and 0.1% FA), eluted at 250 nl/min flow rate with an increasing acetonitrile gradient. The mass spectrometer was operated in data-dependent acquisition (DDA) mode, performed full-scan MS1, at mass/charge ratio = 380 to 1600 with a resolution of 120 K, followed by MS2 acquisitions of the 15 most intense ions with a resolution of 15 K and NCE of 27%. Dynamic exclusion was set for 50 s.

LC-MS/MS data were searched against the UniProt human reviewed fasta database (downloaded 2019) using Mascot (Matrix Science, v2.4), with a precursor tolerance of 10 ppm and a fragment ion mass tolerance of 0.1 Da. Cysteine carbamidomethylation was set as fixed modification, and methionine oxidation, serine, threonine, and tyrosine phosphorylation were specified as variable modifications. MS/MS data were validated using the Scaffold program (Proteome Software Inc., v 4.8.4).

### Preparation of TRF1_core_-teloNCP complex for EM studies

TeloNCP at a final concentration of 3 μM was mixed with TRF1_core_ complex at a final concentration of 30 μM and dialyzed into 25 mM Hepes-KOH (pH 8.0), 150 mM KCl, 1 mM MgCl_2_, 0.01% Igepal CA 630, and 1 mM DTT for 1 hour at 4°C. The sample was loaded onto a 10 to 30% (w/v) glycerol gradient and spun for 16 hours at 50,000 rpm in a SW60 Ti rotor (Beckman Coulter) at 4°C. Two hundred microliters of fractions was collected manually and analyzed by SDS-PAGE. Fractions containing the complex were pooled and cross-linked with 0.5 mM BS3 (Thermo Fisher Scientific) for 1 hour on ice in the dark. After quenching with quench buffer [50 mM tris-HCl (pH 7.4), 150 mM KCl, and 1 mM MgCl_2_], the sample was concentrated and buffer-exchanged into cryo-EM buffer [25 mM Hepes-KOH (pH 8.0), 150 mM KCl, 1 mM MgCl_2_, 1% glycerol, 0.01% Igepal CA-630, and 1 mM DTT].

### Negative stain sample preparation and data collection

Four microliters of the cross-linked TRF1_core_-teloNCP complex from the gradient was applied onto 400-mesh copper grids (Electron Microscopy Sciences, catalog no. G400-Cu) coated with a layer of homemade carbon film on nitrocellulose, which had been glow-discharged for 15 s at 30 mA with a Sputter Coater discharger (Edwards S150B). Following 1.5-min incubation, the grid was incubated with 2% (w/v) uranyl format for a total of 1 min. Data collection was performed using EPU (Thermo Fisher Scientific) on a 200-kV F20 Technai transmission electron microscope equipped with a Falcon II direct electron detector in linear mode with a physical pixel size of 2.02 Å/pixel and with a total dose of 66 electron/Å^2^ over an exposure time of 1.49 s. A dataset of 788 micrographs was collected.

### Negative stain data processing

All data processing described here and subsequent sections was done using RELION 4.0 (*47*, *48*) unless otherwise stated. Contrast transfer function (CTF) parameters were estimated using CTFFIND-4.1 within RELION (*49*). A total of 358,729 particles were picked by RELION LoG picker, binned by 4, and extracted with a 40^2^ pixel box, followed by multiple rounds of reference-free 2D classification to remove junk particles. A subset of 339,645 particles was unbinned and subjected to 25 iterations of 3D classification with an initial angular sample of 7.5°, regularization parameter *T* of 4, and the published map of an unbound nucleosome (EMD-25481) (*50*) as an initial model. The best class of 49,817 particles containing was refined to 15.4-Å resolution. Fitting of the nucleosome structure with two Myb domains bound determined by cryo-EM (see below) shows additional density belonging to TRF1_core_ that is not resolved by cryo-EM.

### Cryo-EM sample preparation and data collection

For cryo-EM sample preparation, grids were first glow-discharged for 70 s at 30 mA using a Sputter coater discharger (model Edwards S150B). Vitrification of grids was performed in liquid ethane at 4°C and 100% humidity using an Vitrobot MK IV (Thermo Fisher Scientific). For TeloNCP, 3 μl of sample was applied onto Quantifoil R1.2/1.3 Au 300-mesh grids (Quantifoil), followed by blotting with Whatman blotting paper (grade 1) at a blot force of −20 for 1.5 s and vitrification. For TRF1_core_-teloNCP complex, 3 μl of sample was applied onto C-flat-T-50 1.2/1.3 grids (Electron Microscopy Sciences, catalog no. CF-1.2/1.3-4Cu-T50), followed by blotting at a blot force of −15 for 2.5 s and vitrification.

Grids were loaded onto a Thermo Fisher Scientific Titan Krios transmission electron microscope operating at 300 kV and equipped with a Gatan K3 direct electron detector camera and a GIF Quantum energy filter. Automatic collection was performed using EPU software (Thermo Fisher Scientific), with the K3 detector in counting mode. For the teloNCP, a total of 12,126 movies were collected at a physical pixel size of 0.73 Å/pixel, with a total electron dose of 40 electrons per Å^2^ over a total exposure time of 1.15 s. Doses were fractionated into 40 movie frames. For the TRF1_core_-teloNCP complex, a total of 24,566 movies were collected at a physical pixel size of 0.826 Å/pixel, with a total electron dose of approximately 56 electrons per Å^2^ over a total exposure time of 2.25 s. Doses were fractionated into 56 movie frames. Slit width of 20 eV on the energy filter and a defocus range of −1 to −2.5 μm were used.

### Cryo-EM data processing

Movie frames were gain-corrected, drift-corrected, dose-weighted, and summed into single micrographs using the motion-correction program implemented within RELION 4.0 (*51*). CTF parameters were estimated for the motion-corrected micrographs using CTFFIND-4.1 (*49*), integrated within RELION.

For the teloNCP dataset (fig. S1, C to E), we used Topaz general model (*52*) for particle picking and selected a total of 958,563 particles with a figure-of-merit (FOM) cutoff value of 0 for further processing. Picked particles were binned by 6, extracted using a box size of 50^2^ pixel, and subjected to a round of 2D classification. A subset of 686,500 particles from selected 2D classes underwent 3D classification with an initial angular sample of 7.5°, regularization parameter *T* of 4 to further remove junk particles. A selected subset of 662,689 particles were unbinned and refined to 3.5-Å resolution. We then performed Bayesian Polishing and refinement of beam tilt, anisotropic magnification, and defocus (*51*, *53*) on this subset of particles, which improved the resolution of the 3D refinement to 2.9-Å resolution. The angular assignments from this refinement are used for alignment-free 3D classification with a regulation parameter *T* of 10. The best class containing 66,482 particles was subsequently refined to 2.5-Å resolution (figs. S1E and S2, A to D).

For the TRF1_core_-teloNCP dataset (fig. S6, A to C), we picked particles using Topaz general model (*52*). A total of 5,031,654 particles with a FOM cutoff value of −2 were binned by 6, extracted with a box size of 70^2^ pixel, and subjected to a round of 2D classification. A subset of 3,851,980 particles from selected 2D classes were subjected to a round of 3D classification with an initial angular sample of 7.5°and regularization parameter *T* of 4. The best 3D class with 2,477,844 particles was refined to 3.4-Å resolution, followed by Bayesian polishing, which improved the resolution of the refinement to 3.2 Å. The outcome of this refinement was taken into two separate directions to resolve the 2:1 and 4:1 TRF1_core_:teloNCP complexes separately.

To obtain the 2:1 TRF1_core_:teloNCP complex, we then used the angular assignments from this refinement for alignment-free 3D classification with a global mask and a regularization parameter *T* of 10. The best subset of 423,088 particles was refined to 3.1-Å resolution, followed by another alignment-free 3D classification with a global mask and a regularization parameter *T* of 14 to further remove suboptimal particles. The best class with 372,307 particles was refined to 3.1 Å. Refinement of beam tilt, anisotropic magnification, and defocus improved the resolution of the 3D refinement to 2.8-Å resolution. To further improve the density of the bound TRF1 Myb domains, we performed focused alignment-free 3D classification using a mask on the two Myb domains and a regularization parameter *T* of 150. We selected a subset of 93,463 particles with the best density of the two Myb domains and refined it to 2.7-Å resolution.

To resolve the 4:1 TRF1_core_:teloNCP complex, we performed alignment-free 3D classification using a mask that covers the long-end of the nucleosome which has lower occupancy of Myb domain binding than the short-end. This allowed us to resolve a class of 312,048 particles with strong density of the Myb domains bound to the long-end of the nucleosome. This class was refined to 3.4-Å resolution. Refinement of beam tilt, anisotropic magnification, and defocus improved the resolution of the 3D refinement of this subset of particles to 3.3-Å resolution. We used the angular assignment of this refinement for another round of alignment-free 3D classification with a global mask and a regularization *T* value of 16. We resolved one class of 17,033 particles with Myb domains bound to both ends of the nucleosome, which refined to 6.7-Å resolution.

Reported resolutions were determined by gold-standard Fourier shell correlation (FSC) = 0.143 of two half-maps resulting from 3D refinements of fully independent data half-sets (*54*). FSCs were calculated with a soft mask (*55*). During postprocessing, the maps were corrected for the modulation transfer function of the detector and sharpened with a negative B-factor as listed in table S1. To further validate our maps, we also used the 3D FSC server to determine the directional FSC and sphericity of the maps (*56*). Local resolution was estimated using RELION, and 2D histograms of the Euler angles were calculated using a Python script called angdis.py (available at https://githubhelp.com/Guillawme/angdist). We used version 1.3 of the script that was updated on 30 October 2021.

### CryoSPARC 3D variability analysis

A subset of 423,088 particles was exported as a particle stack from RELION 4.0 for processing in CryoSPARC v4.1.2 (fig. S8A) (*57*). All steps were carried out using the default parameters unless stated otherwise. Nonuniform refinement (*58*) was performed using a consensus reconstruction from RELION as the initial volume. The resulting reconstruction was a mixture of the Apo teloNCP and the TRF1_core_-teloNCP complexes. Therefore, the particles were subjected to focused 3D classification into six classes, using a mask around the TRF1 Myb domains. The three best TRF1_core_-teloNCP classes with 208,619 particles were grouped and subjected to nonuniform refinement to yield a 3.05-Å reconstruction. 3D variability analysis was then performed by selecting for three modes, using a low-pass filter resolution of 5 Å. The two extreme states are shown in fig. S8A and as a movie showing the morph between the states is shown as movie S1 in the Supplementary Materials.

### Cryo-ET data collection

Tilt series were acquired on a 200-keV Thermo Fisher Scientific Glacios transmission electron microscope equipped with a Falcon III direct electron detector (Thermo Fisher Scientific) and Volta phase plate (VPP; Thermo Fisher Scientific). Single-axis tilt series were recorded using Tomo 5.11 or 5.12 software (Thermo Fisher Scientific) applying a dose-symmetry scheme (*59*) starting at 0°, using a tilt step of 3°, and tilt angles spanning ±60°. Tilt series images with an applied defocus of −2.5 and −3.5 μm and object pixel size of 2.55 and 3.21 Å/pixel, respectively, for apo-nucleosome and bound-nucleosome, were recorded in MRC format on Falcon III operated in linear mode. The exposure time was set to 0.25 and 0.20 s, which provided a flux of 3.10 and 2.90 e^−^/Å^2^, for each tilt image, and a fluence of 127 and 118 e^−^/Å^2^, for the entire tit series of apo-nucleosome and bound-nucleosome, respectively. The data were recorded using the VPP after setting the on-plane conditions, correcting condenser and objective astigmatism, and conditioning the VPP to obtain a ∼π/2 phase shift.

### Tomogram reconstruction and image processing

Tilt series were reconstructed into 3D tomograms by filtered back projection using the Etomo IMOD software package version 4.9.0 (*60*) and binned by a factor of 2 during the process. The tomograms were low-pass–filtered to 5 nm. Further image processing and Z-projecting were performed to obtain the sum of the top, middle, and bottom slices in Fiji (ImageJ2 version 2.9.0/1.53 t). Tomogram surface rendering was performed in Chimera (*61*).

### Model building and refinement

Model building was done in COOT (*62*). To facilitate model building, we converted all maps from MRC format into MTZ format using REFMAC5.8 (*63*) to allow map blurring and sharpening in COOT. The histone octamer and TRF1 Myb domains were rebuilt using PDB 6KE9 and 1W0T as initial models, respectively (*11*, *16*). Because of a shift in DNA register and differences in DNA geometry compared to the published model (PDB 6KE9), we built the DNA model de novo for both the teloNCP and TRF1_core_-teloNCP structures. Model refinement was first performed using Phenix in real-space (*64*) followed by REFMAC5.8 (*63*) in reciprocal space with protein secondary structure restraints and nucleic acid restraints calculated by PROSMART (*65*) and LIBG (*66*), respectively. FSC model versus map were calculated using Phenix (*67*), and geometries are assessed using MolProbity server (*68*) (table S1). Table S2 provides a summary of the refined models.

### Visualization of maps, models, and sequences

Maps and models were visualized using Chimera, ChimeraX (*61*, *69*), and PyMOL (www.pymol.org). Illustrations were prepared using Adobe Illustrator. Sequences were obtained from UniProt, aligned using Clustal Omega server (*70*), and visualized using ESpript 3.0 (*71*).

## Supplementary Materials

### This PDF file includes:

Figs. S1 to S13 Tables S1 and S2 Legend for movie S1 Legends for data S1 to S3 References
