# Supplementary material for: Structural basis of telomeric nucleosome recognition by shelterin factor TRF1
Source: Sci Adv. 2023 Aug 25;9(34):eadi4148. doi: 10.1126/sciadv.adi4148 (PMC10456876; doi:10.1126/sciadv.adi4148)
Supplement: Supplementary file 1 — Figs. S1 to S13 Tables S1 and S2 Legend for movie S1 Legends for data S1 to S3 References [file sciadv.adi4148_sm.pdf]

Science Advances
AAAS

Supplementary Materials for

Structural basis of telomeric nucleosome recognition by shelterin factor TRF1

Hongmiao Hu *et al.*

Corresponding author: Thi Hoang Duong Nguyen, knguyen@mrc-lmb.cam.ac.uk

Sci. Adv. **9**, eadi4148 (2023)
DOI: 10.1126/sciadv.adi4148

The PDF file includes:

Figs. S1 to S13
Tables S1 and S2
Legend for movie S1
Legends for data S1 to S3
References

Other Supplementary Material for this manuscript includes the following:

Movie S1
Data S1 to S3

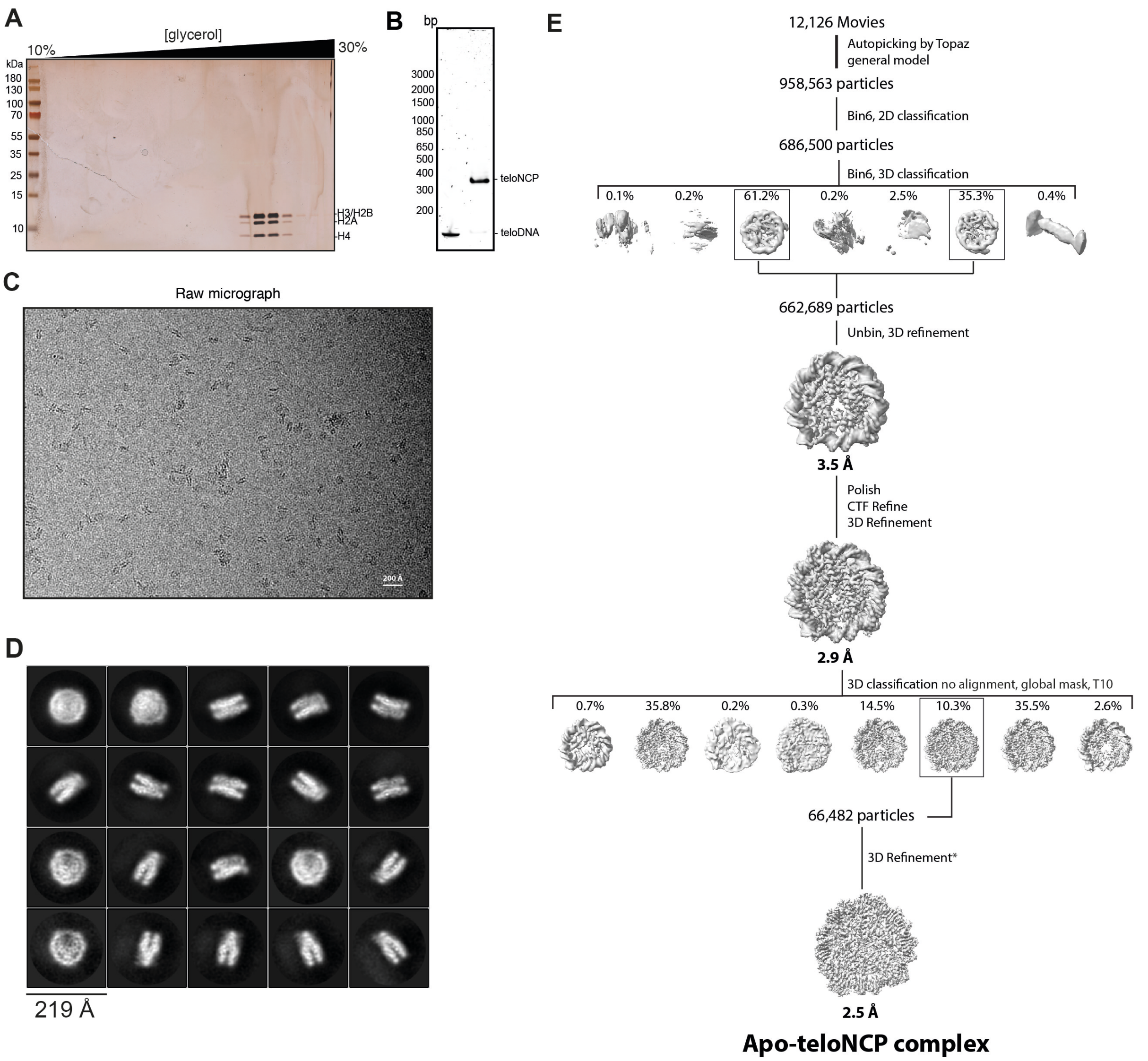

Fig. S1. Reconstitution and cryo-EM structure determination of human telomeric nucleosome

(**A**) Silver-stained SDS-PAGE analysis of fractions from a 10-30% glycerol gradient of the human teloNCP. Peak fractions were subsequently used for structure determination. (**B**) Native gel electrophoresis analysis of the teloNCP compared to the DNA alone. (**C**) and (**D**) Representative cryo-EM image and cryo-EM 2D class averages of the human teloNCP, respectively. (**E**) Cryo-EM data processing strategy for the human teloNCP.

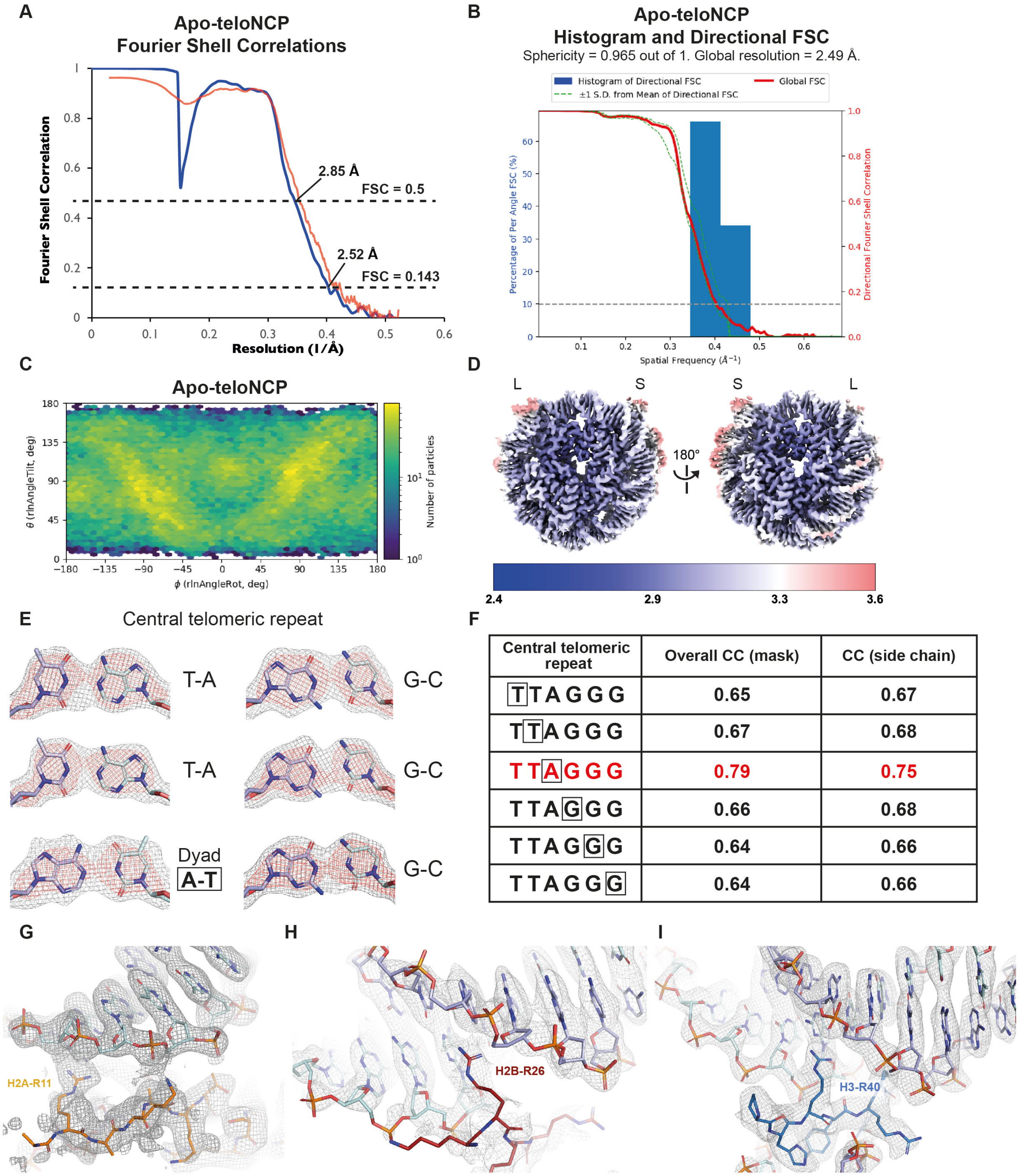

Central telomeric repeat	Overall CC (mask)	CC (side chain)
TTAGGG	0.65	0.67
TTAGGG	0.67	0.68
TTAGGG	0.79	0.75
TTAGGG	0.66	0.68
TTAGGG	0.64	0.66
TTAGGG	0.64	0.66

Fig. S2. Overall and local resolution and representative densities of the 2.5 Å cryo-EM reconstruction of human teloNCP.

(**A**) Gold-standard (blue) and model-vs-map (red) FSC plots for the teloNCP reconstruction. Gold-standard and model-vs-map resolutions were estimated at FSC = 0.143 and 0.5, respectively. (**B**)

Directional FSC plots and sphericity value for the teloNCP reconstruction. Calculations were done using a 3D-FSC server (https://3dfsc.salk.edu/). (**C**) 2D histogram of the Euler angles covered by the particles in the final reconstruction. (**D**) Local resolution estimation of the cryo-EM reconstruction of the teloNCP. This also shows the short end of the nucleosomal DNA is at lower resolution than the long end. (**E**) Cryo-EM density of the central TTAGGG telomeric repeat. Grey and red colored meshes represent low and high contour levels of the map, respectively. The A-T base-pair at the dyad is indicated with a box. (**F**) Model-vs-map cross-correlation (CC) values for models with each of the 6 possible options of DNA positioning on telomeric DNA sequence. The black boxes mark the dyad position on the central telomeric repeat of these sequences. The assigned DNA register in this work is highlighted in red. (**G**), (**H**) and (**I**), Cryo-EM densities in regions where base-specific interactions between DNA and histone H2A, H2B and H3, respectively, are observed (see also Fig. 1B).

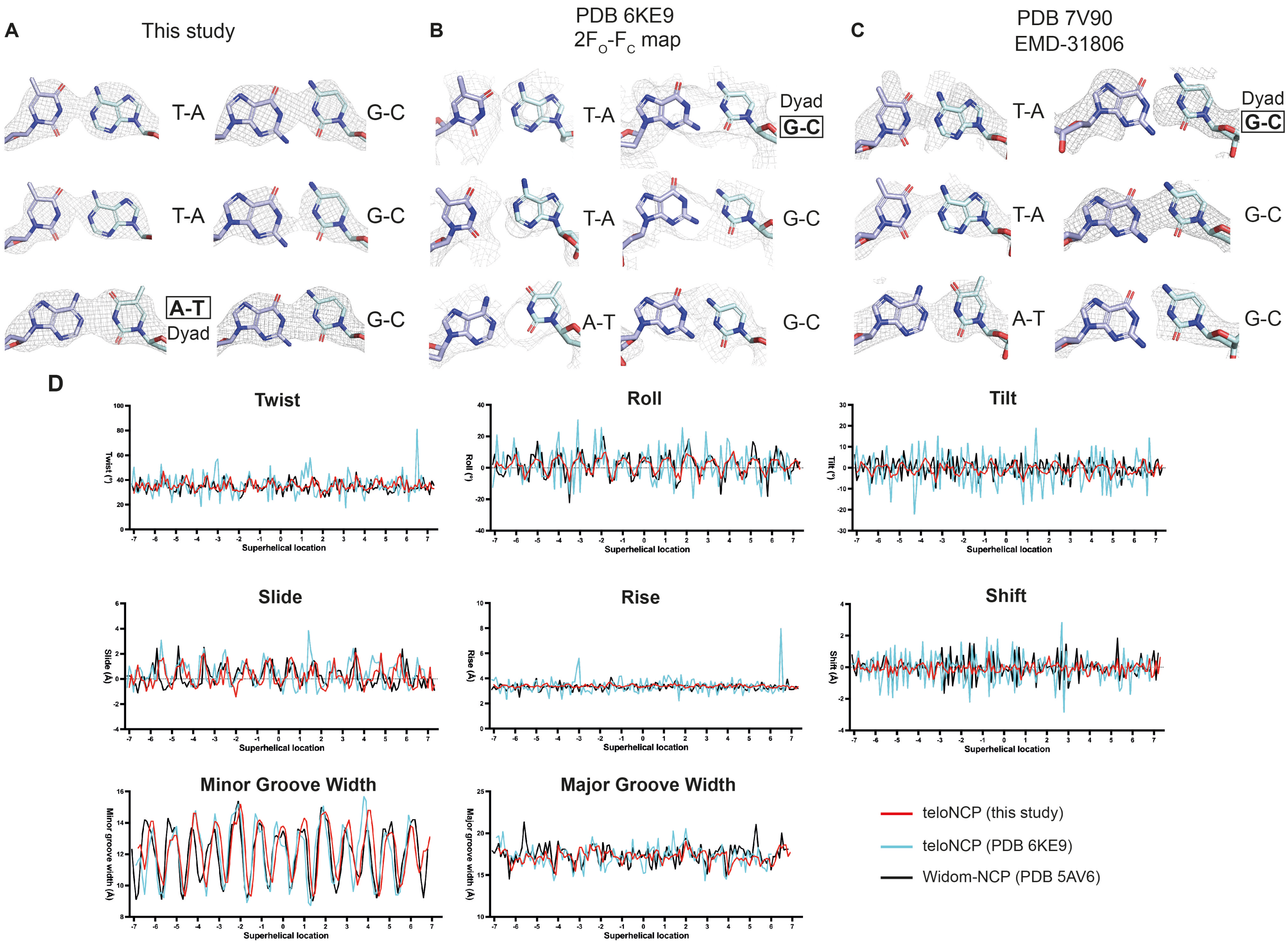
A
This study
T-A
G-C
T-A
G-C
A-T
Dyad
G-C
B
PDB 6KE9
2Fo-Fc map
T-A
Dyad
G-C
T-A
G-C
A-T
G-C
C
PDB 7V90
EMD-31806
T-A
Dyad
G-C
T-A
G-C
A-T
G-C
D
Twist
Twist (°)
Superhelical location
Roll
Roll (°)
Superhelical location
Tilt
Tilt (°)
Superhelical location
Slide
Slide (Å)
Superhelical location
Rise
Rise (Å)
Superhelical location
Shift
Shift (Å)
Superhelical location
Minor Groove Width
Minor groove width (Å)
Superhelical location
Major Groove Width
Major groove width (Å)
Superhelical location
teloNCP (this study)
teloNCP (PDB 6KE9)
Widom-NCP (PDB 5AV6)

Fig. S3. Comparison of our teloNCP structure with the published nucleosome structures

(**A**) Cryo-EM density of the DNA base-pairs in the central telomeric repeat in our 2.5 Å teloNCP structure. The base-pair at the dyad position is boxed and labeled. (**B**) $2F_o$-F_c map of the DNA base-pairs in the central telomeric repeat in the published 2.2 Å teloNCP crystal structure (PDB 6KE9) (*16*). The base-pair at the dyad position is boxed and labeled. (**C**) Cryo-EM density of the DNA base-pairs in the central telomeric repeat in published 3.5 Å teloNCP structure (PDB 7V90, EMD-31806) (*17*). The base-pair at the dyad position is boxed and labeled. (**D**) Comparisons of DNA geometry parameters (twist, rise, roll, slide, tilt, shift, minor groove width and major groove width) of the teloNCP determined in this study (red), the published teloNCP (PDB 6KE9, cyan) (*16*) and NCP reconstituted with the Widom 601 DNA (Widom-NCP) (PDB 5AV6, black) (*18*). The DNA geometries in this study show less distortion and more similarities to the Widom-NCP than the published teloNCP structure. We used the 3DNA server for these geometry analyses (http://web.x3dna.org/) (*72*).

.

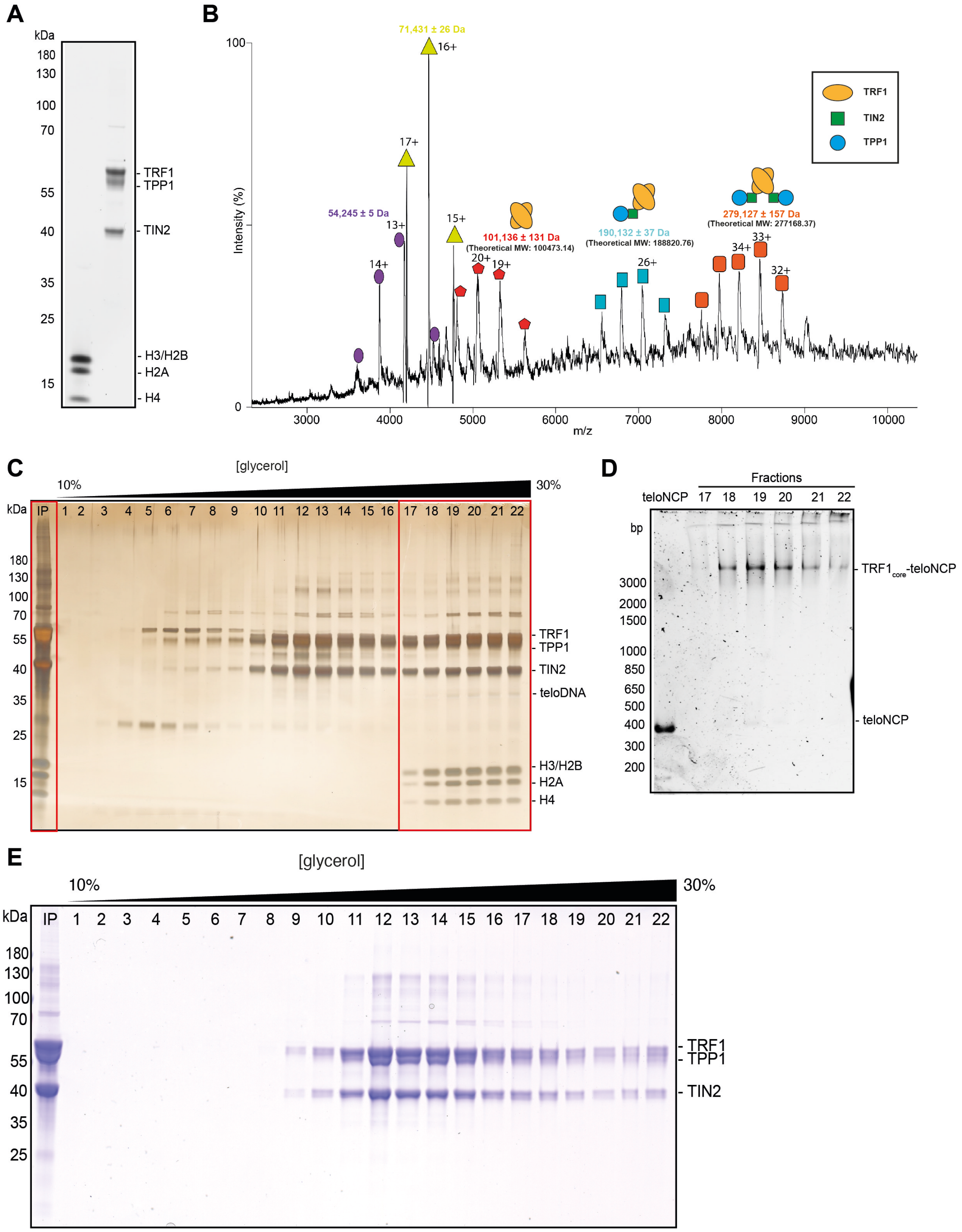

Fig. S4. Reconstitution of the TRF1$_{core}$-teloNCP complex

(**A**) Coomassie-stained SDS-PAGE analysis of purified individual teloNCP and TRF1$_{core}$ complexes. (**B**) Native mass spectrum of recombinant TRF1$_{core}$ complex and subunit composition of assigned peak series. These experiments showed that the TRF1$_{core}$ complex dissociates into

smaller subcomplexes during ionization. Standard deviation represents the error in fitting the identified peaks to the charge series in the measured mass for a single measurement, not error in the mass measurement. Measurements were performed in triplicate with similar results. The 54 and 71 KDa species are contaminants. (**C**) Silver-stained SDS-PAGE analysis of fractions from glycerol gradient purification of the $TRF1_{core}$-teloNCP complex. The left lane shows the input (IP) sample prior to the gradient. Fractions boxed in red (17-22) are those where $TRF1_{core}$ and teloNCP co-migrate on the gradient. (**D**) Native gel analysis of fractions 17-22 from the gradient centrifugation run shown in (C). TeloNCP alone is shown in the left lane as a reference. (**E**) Coomassie SDS-PAGE analysis of fractions from glycerol gradient purification of the $TRF1_{core}$ complex alone.

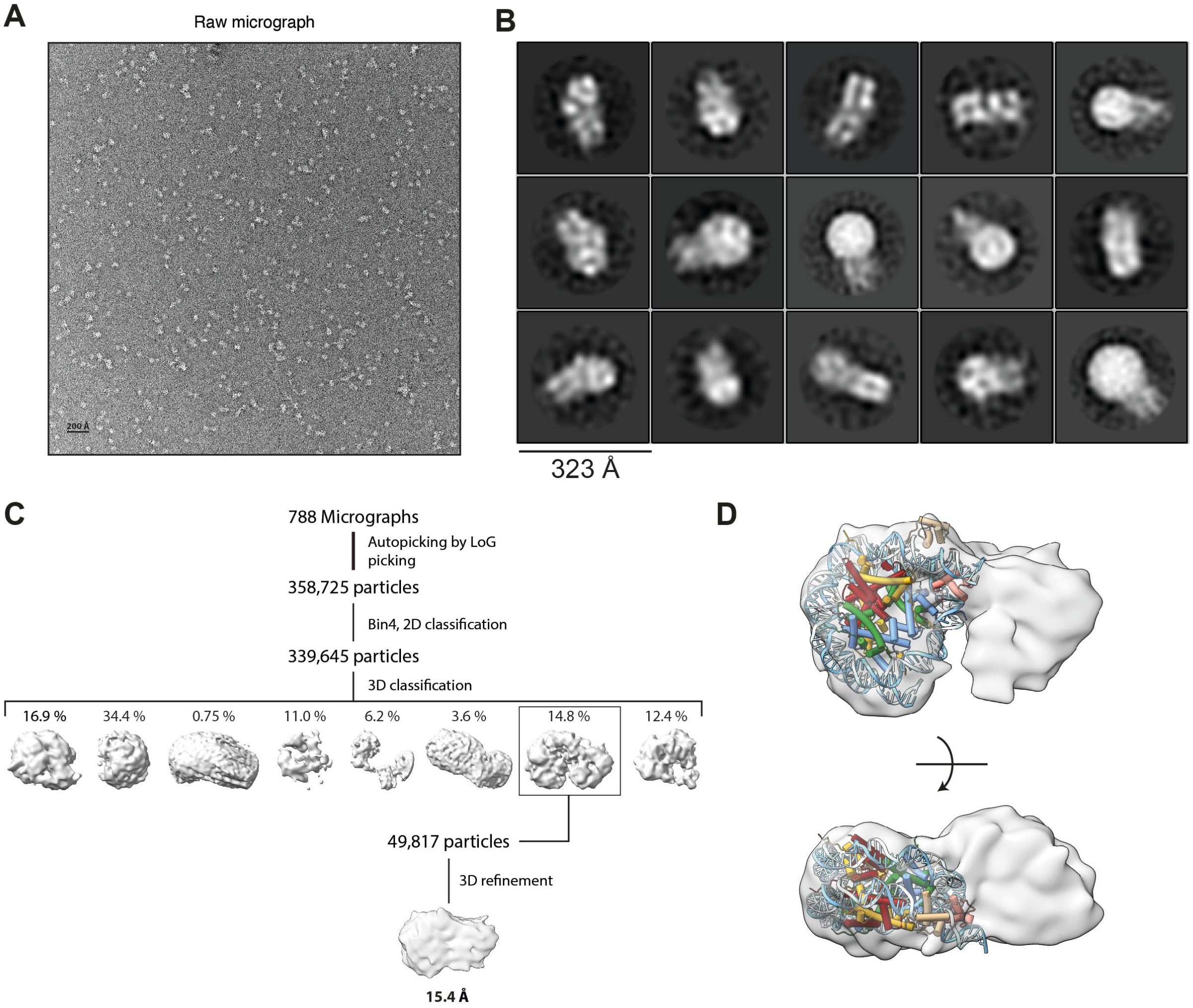

Fig. S5. Negative stain EM reconstruction of the TRF1$_{core}$-teloNCP complex

(**A**) Representative negative stain EM image and (**B**) 2D class averages of the TRF1$_{core}$-teloNCP complex. (**C**) Negative stain EM data processing strategy for the TRF1$_{core}$-teloNCP complex. (**D**) Negative stain reconstruction of the TRF1$_{core}$-teloNCP complex at 15.4 Å resolution. The cryo-EM model of the teloNCP bound to TRF1 fitted in the density show the additional density in the negative stain reconstruction. The missing density in the cryo-EM reconstruction likely results from denaturation at the air-water interface.

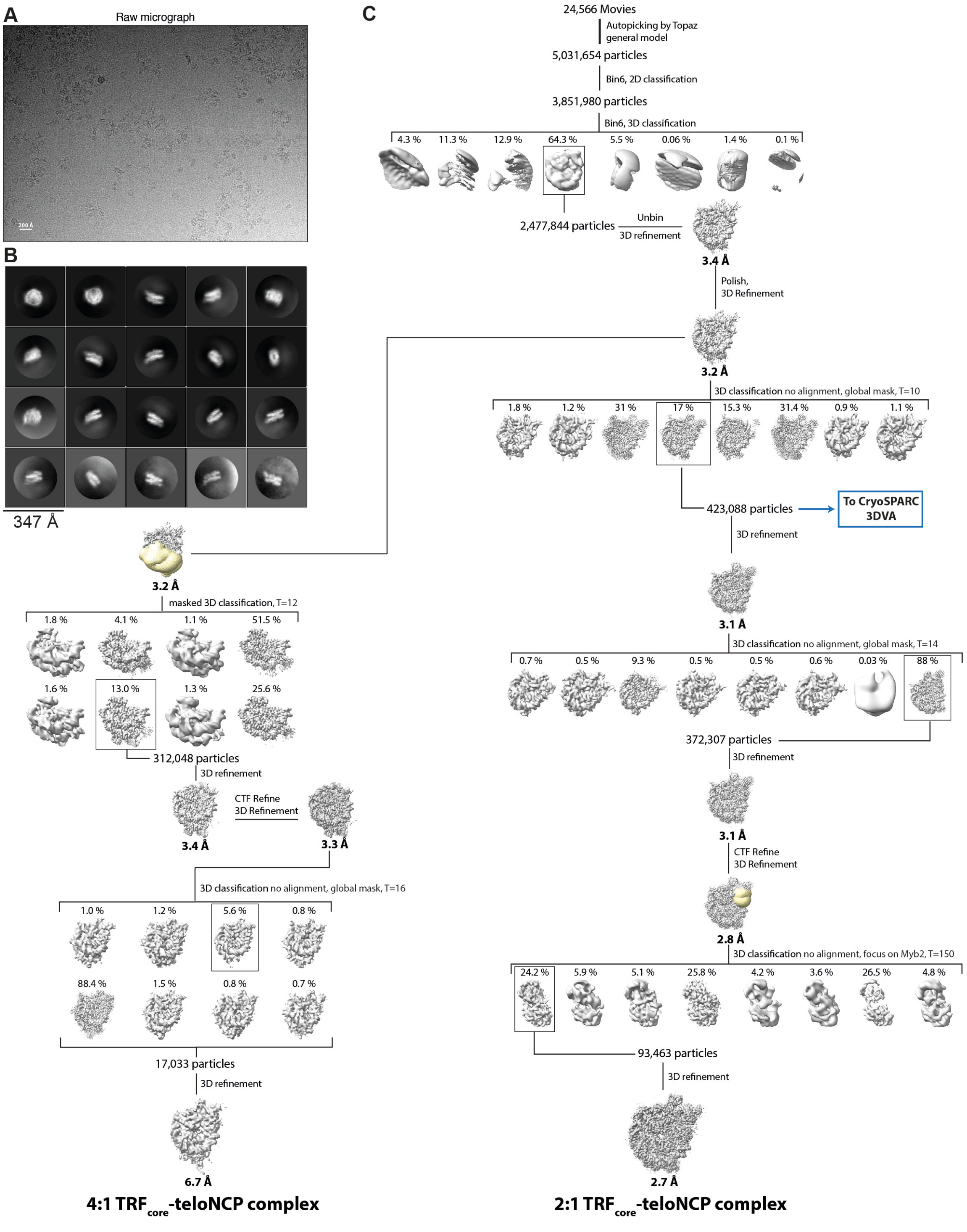

Fig. S6. Cryo-EM data collection and image processing of the TRF1$_{core}$-teloNCP complex

(**A**) Representative cryo-EM image and (**B**) 2D class averages of the TRF1$_{core}$-teloNCP complex. (**C**) Cryo-EM data processing strategy for the TRF1$_{core}$-teloNCP complex.

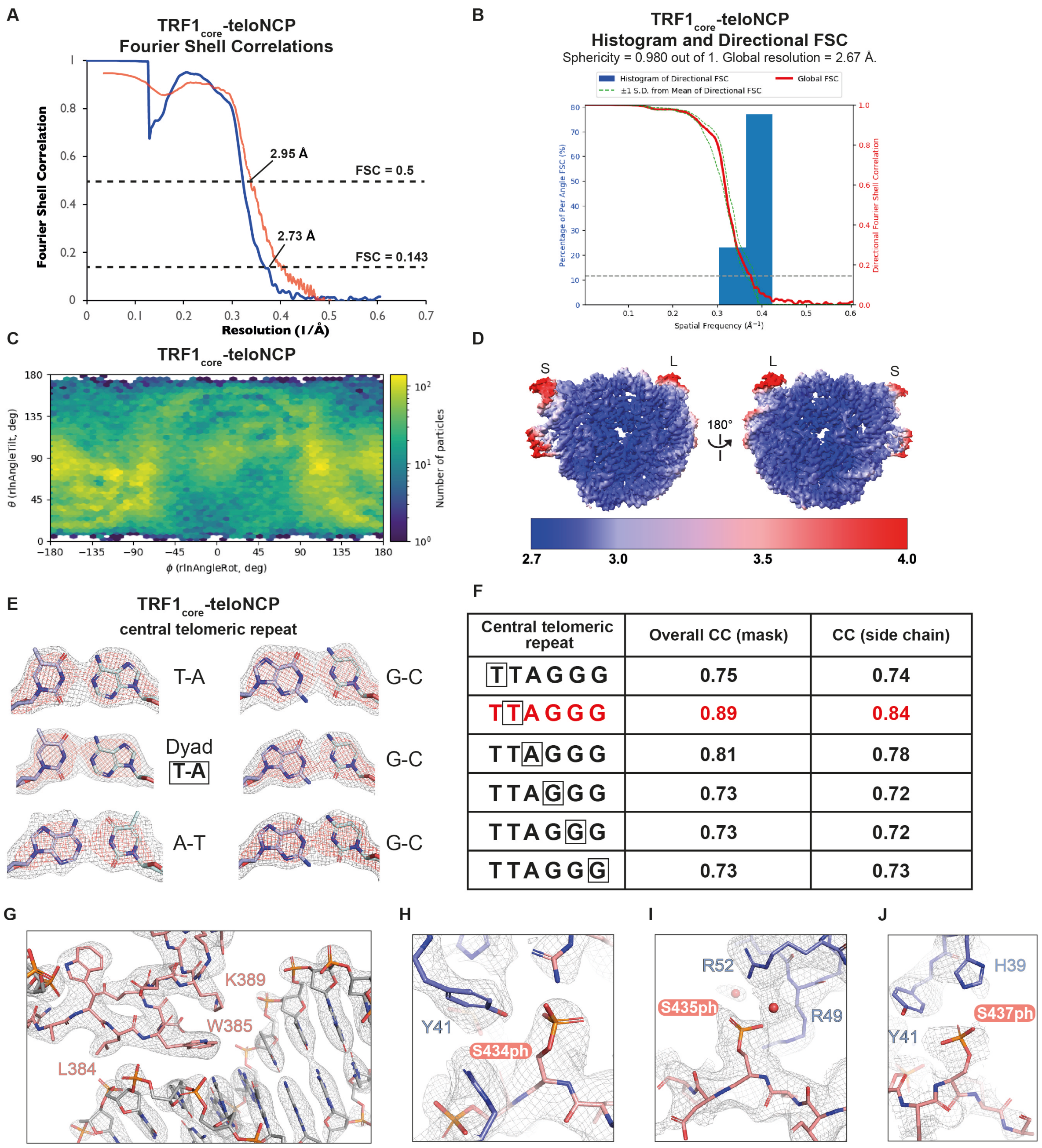

Central telomeric repeat	Overall CC (mask)	CC (side chain)
TTAGGG	0.75	0.74
TTAGGG	0.89	0.84
TTAGGG	0.81	0.78
TTAGGG	0.73	0.72
TTAGGG	0.73	0.72
TTAGGG	0.73	0.73

Fig. S7. Overall and local resolution and representative densities of the 2.7 Å cryo-EM reconstruction of the TRF1$_{core}$-teloNCP complex.

(**A**) Gold-standard (blue) and model-vs-map (red) FSC plots for the TRF1$_{core}$-teloNCP reconstruction. Gold-standard and model-vs-map resolutions were estimated at FSC = 0.143 and 0.5, respectively. (**B**) Directional FSC plots and sphericity value for the TRF1$_{core}$-teloNCP reconstruction. Calculations were done using a 3D-FSC server (https://3dfsc.salk.edu/). (**C**) 2D histogram of the Euler angles covered by the particles in the final reconstruction. (**D**) Local resolution estimation of the density. (**E**) Cryo-EM density of the central TTAGGG telomeric repeat. Grey and red colored meshes represent low and high contour levels of the map,

respectively. The T-A base-pair at the dyad is indicated with a box. (**F**) Model-vs-map cross-correlation (CC) values for models with each of the 6 possible options of DNA positioning on telomeric DNA sequence. The black boxes mark the dyad position on the central telomeric repeat of these sequences. The assigned DNA register in this work is highlighted in red. (**G**) Cryo-EM density showing the interactions between residues in Helix 1 of Myb2 domain and DNA (also see Fig. 4C). (**H**), (**I**) and (**J**), Cryo-EM density of phosphorylated S434, S435 and S437, respectively (see also Fig. 4, B and D).

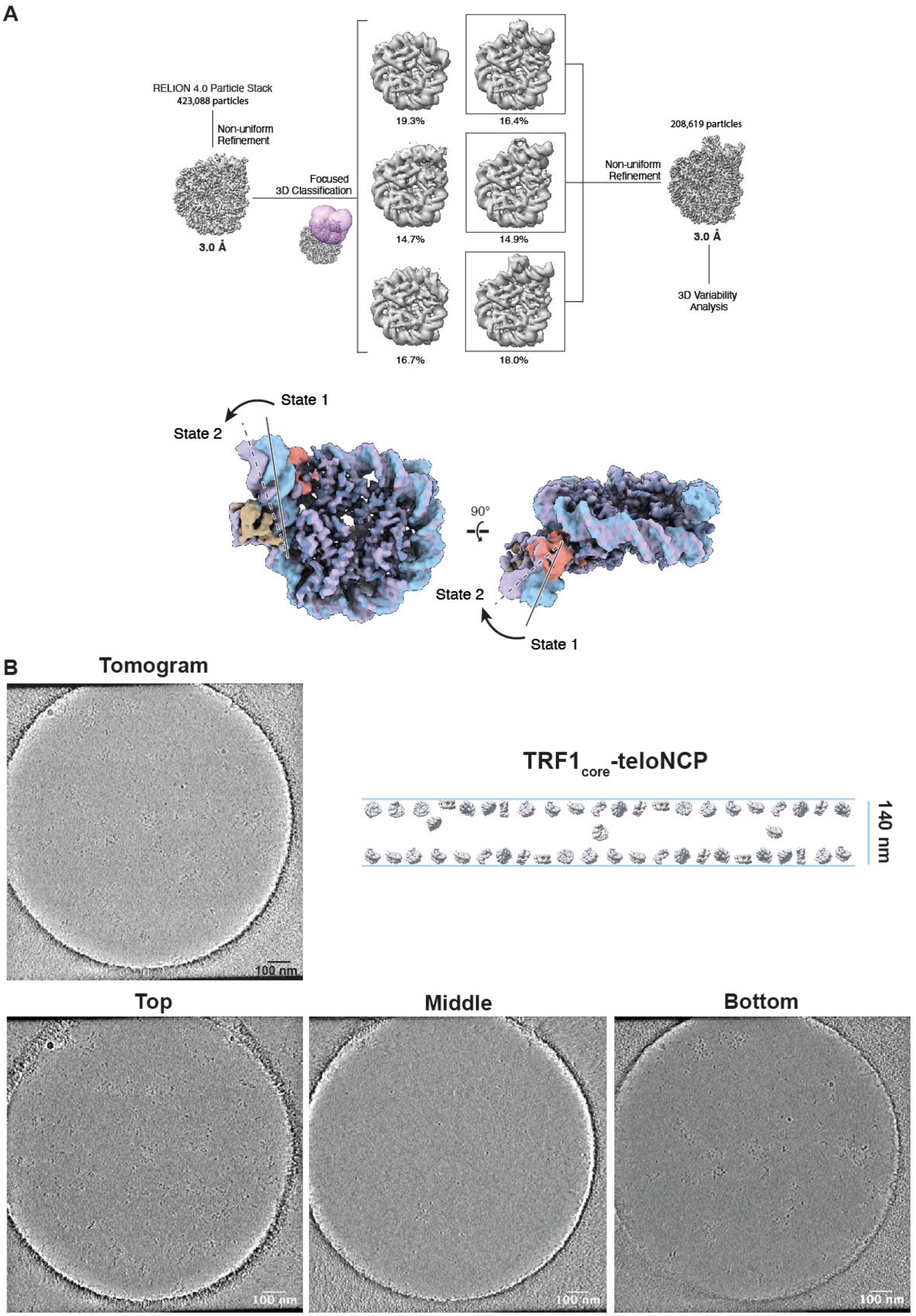

A
RELION 4.0 Particle Stack
423,088 particles
Non-uniform Refinement
3.0 Å
Focused 3D Classification
19.3%
16.4%
14.7%
14.9%
16.7%
18.0%
Non-uniform Refinement
208,619 particles
3.0 Å
3D Variability Analysis
State 1
State 2
90°
State 2
State 1
B
Tomogram
100 nm
TRF1core-teloNCP
140 nm
Top
Middle
Bottom
100 nm
100 nm
100 nm

Fig. S8. 3D variability analysis and cryo-electron tomographic analysis of particle distribution on the cryo-EM grid of the TRF1$_{core}$-teloNCP complex.

(**A**) Summary of the processing strategy for 3DVA of a selected subset of particles (see fig. S6) in CryoSPARC (*58*) (top). The indicated subsets with clear Myb domain density were subjected to refinement followed by 3DVA. Comparison of the 3DVA conformations (bottom, State 1 and State 2). The first and last frames from the first orthogonal principle mode are shown (State 1 and State 2, respectively). Curved arrows indicate the degree of variability in State 2 relative to State 1. The results are also summarized in Movie S1. (**B**) The z-projected sum of the tomogram slices of TRF1$_{core}$-teloNCP complex. The z-projected sum of the top, middle and bottom slices of the tomograms are shown in the bottom inset, whereas cross-sectional schematic diagrams are shown on right-hand insets depicting particle distribution in ice within the holes.

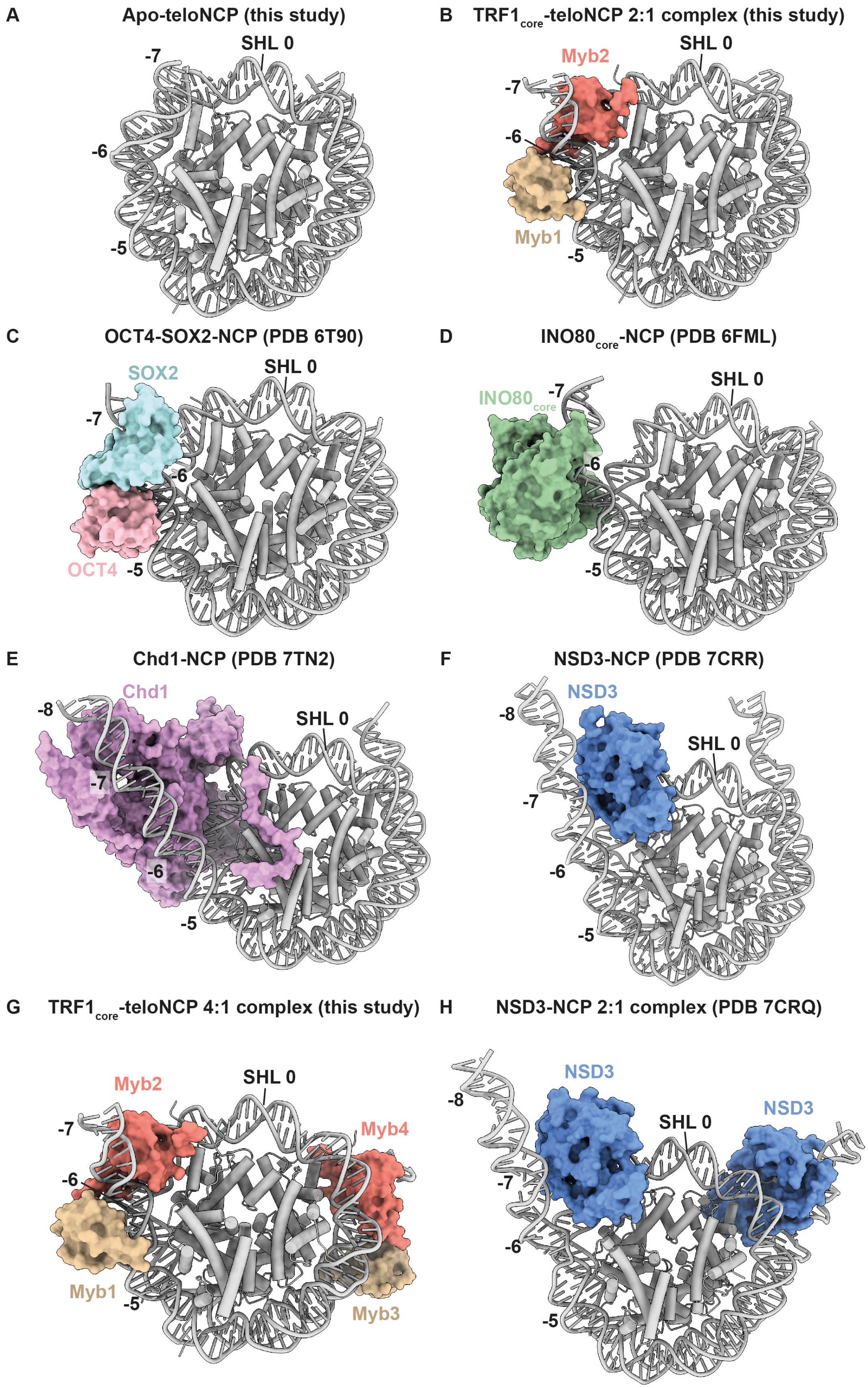
A
Apo-teloNCP (this study)
SHL 0
-7
-6
-5
B
TRF1core-teloNCP 2:1 complex (this study)
Myb2
SHL 0
-7
-6
Myb1
-5
C
OCT4-SOX2-NCP (PDB 6T90)
SOX2
SHL 0
-7
-6
OCT4
-5
D
INO80core-NCP (PDB 6FML)
INO80core
-7
SHL 0
-6
-5
E
Chd1-NCP (PDB 7TN2)
Chd1
-8
SHL 0
-7
-6
-5
F
NSD3-NCP (PDB 7CRR)
NSD3
-8
SHL 0
-7
-6
-5
G
TRF1core-teloNCP 4:1 complex (this study)
Myb2
SHL 0
-7
Myb4
-6
Myb1
-5
Myb3
H
NSD3-NCP 2:1 complex (PDB 7CRQ)
NSD3
-8
SHL 0
NSD3
-7
-6
-5

Fig. S9. Structure comparison with other nucleosome binding proteins/complexes

(**A**), (**B**) Structures of the apo-teloNCP and 2:1 $TRF1_{core}$-teloNCP complex obtained in this study, respectively. (**C**) Structure of nucleosome core particles (NCP) with pioneer transcription factors OCT4 and SOX2 (PDB 6T90) (*40*). (**D**) Structure of NCP with $INO80_{core}$ chromatin remodeler (PDB 6FML) (*73*). (**E**) Structure of NCP with Chd1 chromatin remodeler (PDB 7TN2) (*50*). (**F**) Structure of NCP with nuclear receptor-binding SET domain protein 3 (NSD3) methyltransferase (PDB 7CRR) (*25*). (**G**) Structure of 4:1 $TRF1_{core}$-teloNCP complex obtained in this study. (**H**) Structure of 2:1 NSD3 methyltransferase-NCP complex (PDB 7CRQ) (*25*). In all figures, the histones and DNA are colored in grey and nucleosome binding proteins are colored as indicated.

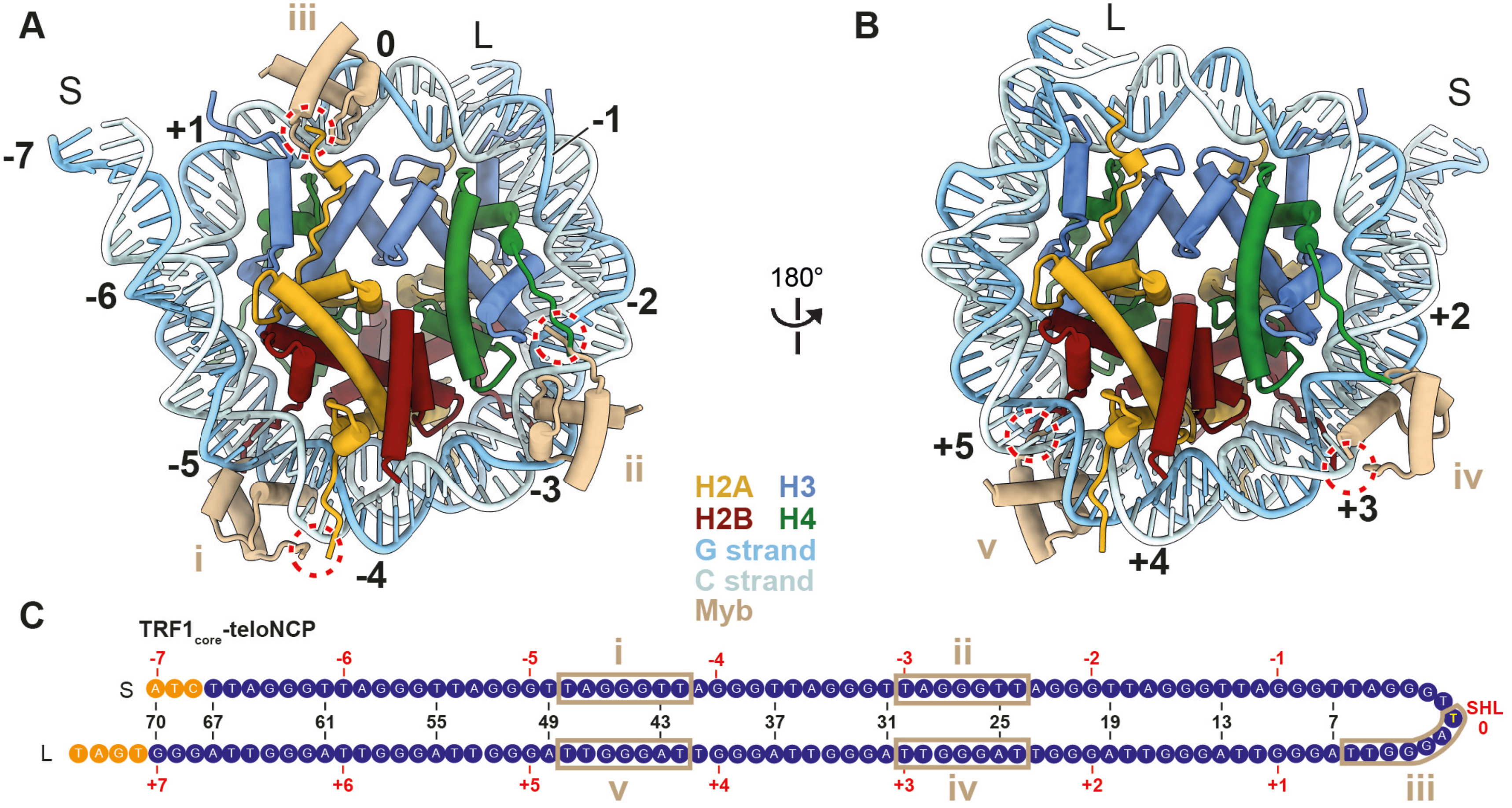

Fig. S10. Alternative TRF1 Myb domain binding sites on the nucleosome

(**A**), (**B**) Front and back views of the teloNCP with TRF1 Myb domain binding to 5 alternative sites (labelled i-v) on the telomeric nucleosomal DNA, respectively. Clashes with histone tails are highlighted in red circles. Only exposed binding sites on the outer gyres are modelled. (**C**) DNA sequence used in this study with the alternative binding sites indicated in wheat colored boxes.

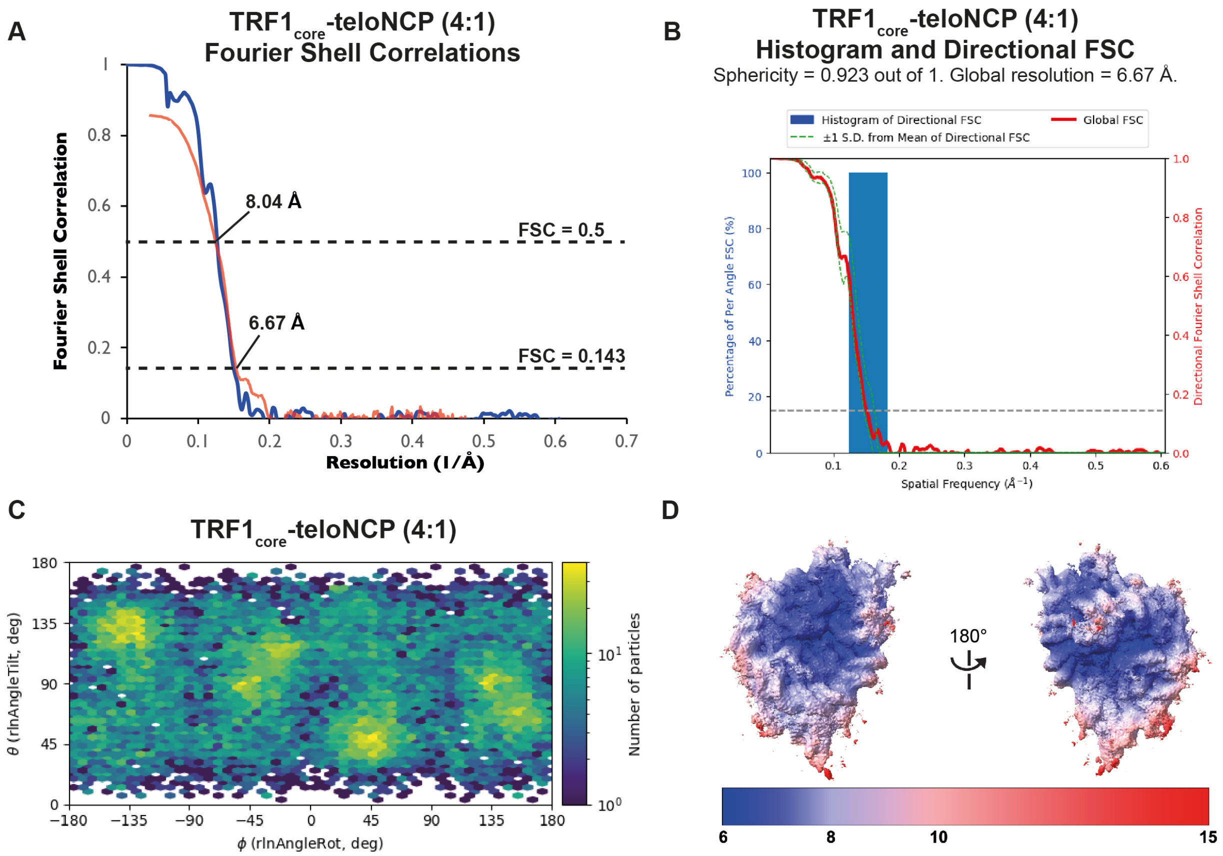

Fig. S11. Overall and local resolution and representative densities of the 6.7 Å cryo-EM reconstruction.

(**A**) Gold-standard (blue) and model-vs-map (red) FSC plots for the TRF1$_{core}$-teloNCP 4:1 reconstruction. Gold-standard and model-vs-map resolutions were estimated at FSC = 0.143 and 0.5, respectively. Note that this model was obtained from rigid-body docking and not refined due to the limited resolution of the density. (**B**) Directional FSC plots and sphericity value for the teloNCP reconstruction. Calculations were done using a 3D-FSC server (https://3dfsc.salk.edu/). (**C**) 2D histogram of the Euler angles covered by the particles in the final reconstruction. (**D**) Local resolution estimation of the density.

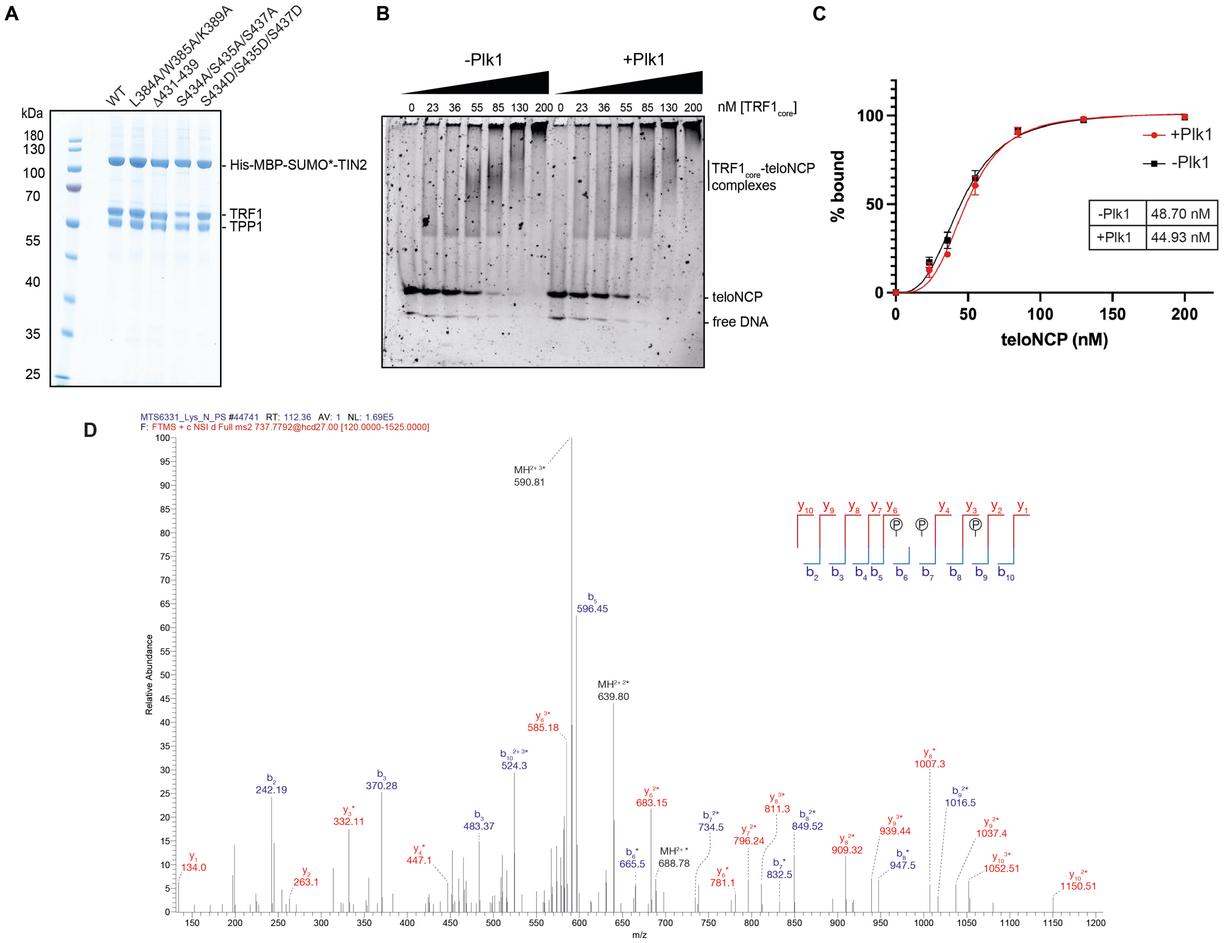

E

Protein	Phosphorylation sites identified
TRF1	S11, S104, S274, S279, S296, S298, S302, T305, S307, S308, S317, T371, S404, S417, S434, S435, S437
TIN2	T3, S90, S295, T297, T320, S324, S328, S330, T334
TPP1	S20, T22, S24, S25, S79, S313, S316, S330, S338, S339, S347, S349, S412

F

Literatures	Phosphorylation sites	Sample for proteomic studies
Wu et al. (*ref 32*)	S435	-
Klammer et al. (*ref 28*)	S435, S437	non-small cell lung cancer
Franz-Wachtel et al. (*ref 29*)	S434, S435, S437	PKD1 active vs. kinase dead mutant
Beli et al. (*ref 30*)	S434, S435, S437	DNA damage response proteome
Weber et al. (*ref 31*)	S434, S435, S437	acute myeloid leukemia cells
Dephoure et al. (*ref 26*)	S434, S435, S437	G1-phase and M-phase proteome
Mertins et al. (*ref 27*)	mouse TRF1 S421	mouse stroma

Fig. S12. Purification of all mutant TRF1$_{core}$ complexes, Plk1 treatment of TRF1$_{core}$ and identification of phosphorylation sites on TRF1$_{core}$ by mass spectrometry.

(**A**) Coomassie-stained SDS-PAGE of wild-type (WT) and the four TRF1$_{core}$ mutants used in EMSA experiments shown in Fig. 4. (**B**) EMSAs showing titration of TRF1$_{core}$ without (-Plk1) and with Plk1 (+Plk1) treatment against teloNCP. (**C**) Quantification of EMSA experiments shown in

panel (B). In the left panel, we plotted percentages of unbound teloNCPs as a function of protein concentration in the EMSA reactions. The right table shows the concentration of the $TRF1_{core}$ complex at which 50% of teloNCP remains unbound as determined from the graphs. Error bars are the standard error of the mean (SEM) obtained from the three replicates. (**D**) Mass spectrum of the C-terminal peptide of TRF1 (residues 429-439) from the LC-MS/MS analysis. Sites of phosphorylation were identified as denoted. The asterisks (*) denote the loss of phosphoric acid (-98) from the precursor and/or product ions that harbour the phosphorylated residues. (**E**) A full list of all phosphorylated sites identified in the $TRF1_{core}$ samples used in this study. (**F**) Summary of previous proteomic studies that have identified phosphorylation of the three serine residues (S434, S435 and S437 in human or equivalent residues in mouse) (*26-32*).

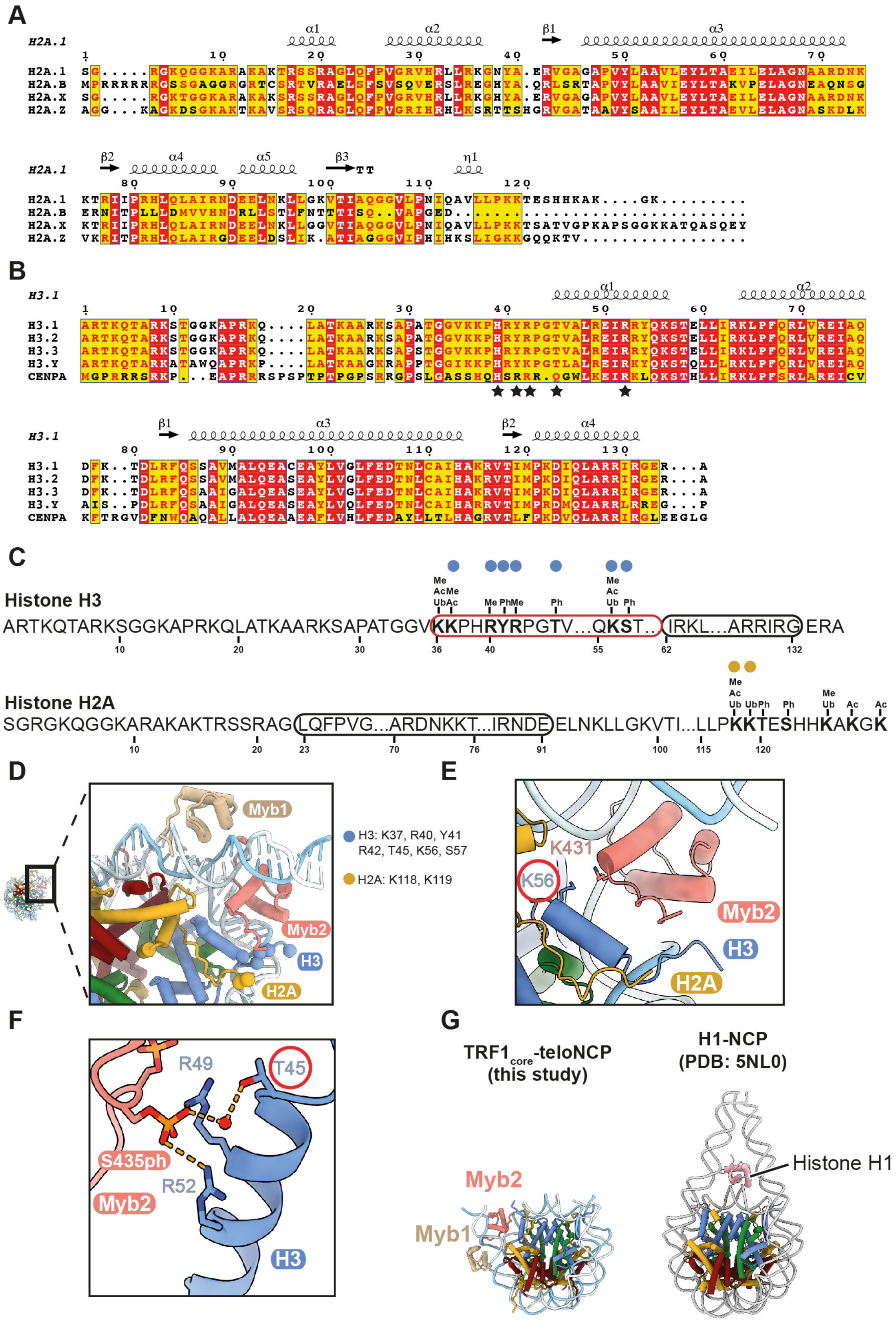
A
B
C
Histone H3
ARTKQTARKSGGKAPRKQLATKAARKSAPATGGVKKPHRYRPGTV...QKST...IRKL...ARRIRGERA
Histone H2A
SGRGKQGGKARAKAKTRSSRAGLQFPVG...ARDNKKT...IRNDELNKLLGKVTI...LLPKKTESHHKAKGK
D
Myb1
Myb2
H3
H2A
H3: K37, R40, Y41 R42, T45, K56, S57
H2A: K118, K119
E
K431
K56
Myb2
H3
H2A
F
R49
T45
S435ph
R52
Myb2
H3
G
TRF1core-teloNCP (this study)
H1-NCP (PDB: 5NL0)
Myb2
Myb1
Histone H1

Fig. S13. Sequence alignment and post-translational modifications of histone H2A and H3 and the incompatibility with histone H1 binding.

(**A**) Sequence alignment of human histone H2A variants. The variant used in this study is H2A.1. (**B**) Sequence alignment of human histone H3 variants. The variant used in this study is H3.1. Residues involved in interactions with TRF1 are indicated with stars underneath the sequence. (**C**) Sequences of histone H3 and H2A highlighting known post-translational modifications (*42*). The black boxes indicate the core histone fold and the red box indicates the region on histone H3, which is in direct contact with TRF1 Myb2 domain. Residues marked with blue and yellow balls on top of the sequences are those with known PTMs and in either direct contact or close proximity with TRF1 in the $TRF1_{core}$-teloNCP structure. (**D**) TRF1 interactions with histone H2A and H3. Blue and yellow balls highlight residues with known PTMs and in either direct contact or close proximity with TRF1 in the $TRF1_{core}$-teloNCP structure. They are also highlighted in (C). (**E**) Close-up view of interaction between residue K431 of TRF1 and K56 of histone H3. Histone H3 K56 is known to bear multiple types of PTMs, which will potentially disrupt this interaction. (**F**) Close-up view of interaction between phosphorylated S435 of TRF1 and residues of histone H3. T45 of histone H3 is known to be phosphorylated, which can also change its interaction with TRF1. (**G**) Comparison of the 2:1 $TRF1_{core}$-teloNCP structure and the histone H1 bound-NCP (PDB 5NL0) (*74*). This shows that binding of TRF1 is incompatible to histone H1 binding.

Table S1. Cryo-EM data collection, refinement, and validation statistics

	teloNCP<br>PDB 8OX0<br>EMD-17251	2:1 $TRF1_{core}$-teloNCP<br>PDB 8OX1<br>EMD-17252	4:1 $TRF1_{core}$-teloNCP<br>EMD-17253
**Data collection and Processing**			
Microscope	TFS Titan Krios	TFS Titan Krios	
Voltage (keV)	300	300	
Camera	Gatan K3	Gatan K3	
Magnification	105,000	105,000	
Pixel size at detector (Å/pixel)	0.73	0.826	
Total electron exposure (e^{-}/Å^2)	40	56	
Exposure rate (e-/ Å^2/sec)	34.8	24.9	
Number of frames	40	56	
Defocus range (µm)	0.8-2.0	1.0-2.5	
Automation software	EPU	EPU	
Energy filter slit width	20 eV	20 eV	
Micrographs collected (no.)	12,126	24,566	
Total extracted particles (no.)	958,563	5,031,654	
**For each reconstruction:**			
Final particles (no.)	66,482	93,463	17,033
Point-group	C1	C1	C1
Estimated error (translations/rotations)	0.51/1.20	0.42/0.93	1.53/2.92
Resolution (global, Å)	2.5	2.7	6.7
FSC 0.5 (unmasked/masked)	3.0/2.9	3.1/3.0	8.0/8.5
FSC 0.143 (unmasked/masked)	3.1/2.5	3.5/2.7	/6.7
Resolution range (local, Å)	2.4-4.0	2.8-4.4	6.2-15.3
3DFSC Sphericity	0.965	0.980	0.923
Map sharpening *B* factor (Å^2)	-30	0	-100
Map sharpening methods	RELION4.0	RELION4.0	RELION4.0
**Model composition**			
Protein (residues)	787	905	1029
RNA/DNA (nucleotides)	290	290	282
**Model Refinement**			
Refinement package	Phenix and REFMAC	Phenix and REFMAC	
- real or reciprocal space	Real/Reciprocal Space	Real/Reciprocal Space	
- resolution cutoff	0.5	0.5	
Model-Map scores			
-CCvolume/mask	0.79/0.79	0.89/0.89	
B factors (Å^2)			
Protein residues (min/max/mean)	88.59/357.98/135.34	42.74/281.95/103.16	
RNA/DNA (min/max/mean)	94.68/355.85/159.85	55.45/290.90/121.42	
R.m.s. deviations from ideal values			
Bond lengths (Å) (#>4σ)	0.007 (0)	0.009 (12)	
Bond angles (°) (#>4σ)	0.890 (0)	1.063 (3)	

**Validation**		
MolProbity score	0.74	1.17
CaBLAM outliers (%)	0.66	0.92
Clashscore	0.74	3.82
Poor rotamers (%)	0	0
C-beta deviations (%)	0	0.12
EMRinger score	4.78	4.59
Ramachandran plot		
Favored (%)	98.96	98.18
Outliers (%)	0	0

Table S2. Summary of modelled protein and DNA

				**teloNCP**<br>PDB 8OX0			**TRF1$_{core}$-teloNCP**<br>PDB 8OX1		
Molecule	Chain ID	Uniprot ID	Total Res.	Modelled Res.	Template Used	Modeling Approach	Modelled Residues	Template Used	Modelling Approach
H2A	C	Q93077	130	10-119	5AV6	Docked & Adjusted	10-119	5AV6	Docked & Adjusted
	G	Q93077	130	10-119	5AV6	Docked & Adjusted	10-119	5AV6	Docked & Adjusted
H2B	D	P62807	166	25-122	5AV6	Docked & Adjusted	25-122	5AV6	Docked & Adjusted
	H	P62807	166	25-122	5AV6	Docked & Adjusted	24-122	5AV6	Docked & Adjusted
H3	A	P68431	136	37-135	5AV6	Docked & Adjusted	37-134	5AV6	Docked & Adjusted
	E	P68431	136	37-134	5AV6	Docked & Adjusted	37-134	5AV6	Docked & Adjusted
H4	B	P62805	103	15-102	5AV6	Docked & Adjusted	20-102	5AV6	Docked & Adjusted
	F	P62805	103	17-102	5AV6	Docked & Adjusted	18-102	5AV6	Docked & Adjusted
Telomeric C strand	I	-	145	-73-71	-	*de novo*	-74-70	-	*de novo*
Telomeric G strand	J	-	145	-71-73	-	*de novo*	-70-74	-	*de novo*
TRF1	L	P54274	439	-	-	-	377-435	1W0T	Docked & Adjusted
	M	P54274	439	-	-	-	375-439	Alphafold2 predicted TRF1 protein	Predicted & Adjusted

Auxiliary Supplementary Materials

Movie S1
Morphs between the 3D volumes generated by 3DVA in cryoSPARC (fig. S8A).

Data S1
Pymol session containing the refined structure of apo-teloNCP complex (Fig. 1A, B; fig. S2).

Data S2
Pymol session containing the refined structure of the 2:1 TRF1core-teloNCP complex (Fig. 2A, B; fig. S7).

Data S3
Pymol session containing the rigid-body fitted structure of the 4:1 TRF1core-teloNCP complex (Fig. 3A,B; fig. S11).

REFERENCES AND NOTES

1. T. de Lange, Shelterin-Mediated Telomere Protection. *Annu. Rev. Genet.* **52**, 223–247 (2018).

2. C. J. Lim, T. R. Cech, Shaping human telomeres: From shelterin and CST complexes to telomeric chromatin organization. *Nat. Rev. Mol. Cell Biol.* **22**, 283–298 (2021).

3. J. W. Shay, Role of Telomeres and Telomerase in Aging and Cancer. *Cancer Discov.* **6**, 584–593 (2016).

4. H. Tommerup, A. Dousmanis, T. de Lange, Unusual chromatin in human telomeres. *Mol. Cell. Biol.* **14**, 5777–5785 (1994).

5. V. L. Makarov, S. Lejnine, J. Bedoyan, J. P. Langmore, Nucleosomal Organization of telomere-specific chromatin in rat. *Cell* **73**, 775–787 (1993).

6. Z. Zhong, L. Shiue, S. Kaplan, T. de Lange, A mammalian factor that binds telomeric TTAGGG repeats in vitro. *Mol. Cell. Biol.* **12**, 4834–4843 (1992).

7. T. Bilaud, C. Brun, K. Ancelin, C. E. Koering, T. Laroche, E. Gilson, Telomeric localization of TRF2, a novel human telobox protein. *Nat. Genet.* **17**, 236–239 (1997).

8. D. Broccoli, A. Smogorzewska, L. Chong, T. de Lange, Human telomeres contain two distinct Myb–related proteins, TRF1 and TRF2. *Nat. Genet.* **17**, 231–235 (1997).

9. A. Bianchi, S. Smith, L. Chong, P. Elias, T. de Lange, TRF1 is a dimer and bends telomeric DNA. *EMBO J.* **16**, 1785–1794 (1997).

10. L. Fairall, L. Chapman, H. Moss, T. de Lange, D. Rhodes, Structure of the TRFH dimerization domain of the human telomeric proteins TRF1 and TRF2. *Mol. Cell* **8**, 351–361 (2001).

11. R. Court, L. Chapman, L. Fairall, D. Rhodes, How the human telomeric proteins TRF1 and TRF2 recognize telomeric DNA: A view from high-resolution crystal structures. *EMBO Rep.* **6**, 39–45 (2005).

12. A. Galati, L. Rossetti, S. Pisano, L. Chapman, D. Rhodes, M. Savino, S. Cacchione, The human telomeric protein TRF1 specifically recognizes nucleosomal binding sites and alters nucleosome structure. *J. Mol. Biol.* **360**, 377–385 (2006).

13. S. Pisano, D. Leoni, A. Galati, D. Rhodes, M. Savino, S. Cacchione, The human telomeric protein hTRF1 induces telomere-specific nucleosome mobility. *Nucleic Acids Res.* **38**, 2247–2255 (2010).

14. A. Galati, E. Micheli, C. Alicata, T. Ingegnere, A. Cicconi, M. C. Pusch, M.-J. Giraud-Panis, E. Gilson, S. Cacchione, TRF1 and TRF2 binding to telomeres is modulated by nucleosomal organization. *Nucleic Acids Res.* **43**, 5824–5837 (2015).

15. M.-J. Giraud-Panis, S. Pisano, D. Benarroch-Popivker, B. Pei, M.-H. Le Du, E. Gilson, One identity or more for telomeres? *Front. Oncol.* **3**, 48 (2013).

16. A. Soman, C. W. Liew, H. L. Teo, N. V. Berezhnoy, V. Olieric, N. Korolev, D. Rhodes, L. Nordenskiöld, The human telomeric nucleosome displays distinct structural and dynamic properties. *Nucleic Acids Res.* **48**, 5383–5396 (2020).

17. A. Soman, S. Y. Wong, N. Korolev, W. Surya, S. Lattmann, V. K. Vogirala, Q. Chen, N. V. Berezhnoy, J. van Noort, D. Rhodes, L. Nordenskiöld, Columnar structure of human telomeric chromatin. *Nature* **609**, 1048–1055 (2022).

18. M. Wakamori, Y. Fujii, N. Suka, M. Shirouzu, K. Sakamoto, T. Umehara, S. Yokoyama, Intra- and inter-nucleosomal interactions of the histone H4 tail revealed with a human nucleosome core particle with genetically-incorporated H4 tetra-acetylation. *Sci. Rep.* **5**, 17204 (2015).

19. H. Ai, M. Sun, A. Liu, Z. Sun, T. Liu, L. Cao, L. Liang, Q. Qu, Z. Li, Z. Deng, Z. Tong, G. Chu, X. Tian, H. Deng, S. Zhao, J. B. Li, Z. Lou, L. Liu, H2B Lys34 ubiquitination induces nucleosome distortion to stimulate Dot1L activity. *Nat. Chem. Biol.* **18**, 972–980 (2022).

20. T. M. Weaver, N. M. Hoitsma, J. J. Spencer, L. Gakhar, N. J. Schnicker, B. D. Freudenthal, Structural basis for APE1 processing DNA damage in the nucleosome. *Nat. Commun.* **13**, 5390 (2022).

21. J. Z.-S. Ye, D. Hockemeyer, A. N. Krutchinsky, D. Loayza, S. M. Hooper, B. T. Chait, T. de Lange, POT1-interacting protein PIP1: A telomere length regulator that recruits POT1 to the TIN2/TRF1 complex. *Genes Dev.* **18**, 1649–1654 (2004).

22. M. S. O'Connor, A. Safari, H. Xin, D. Liu, Z. Songyang, A critical role for TPP1 and TIN2 interaction in high-order telomeric complex assembly. *Proc. Natl. Acad. Sci. U.S.A.* **103**, 11874–11879 (2006).

23. J. C. Zinder, P. D. B. Olinares, V. Svetlov, M. W. Bush, E. Nudler, B. T. Chait, T. Walz, T. de Lange, Shelterin is a dimeric complex with extensive structural heterogeneity. *Proc. Natl. Acad. Sci. U.S.A.* **119**, e2201662119 (2022).

24. K. A. Taylor, R. M. Glaeser, Retrospective on the early development of cryoelectron microscopy of macromolecules and a prospective on opportunities for the future. *J. Struct. Biol.* **163**, 214–223 (2008).

25. W. Li, W. Tian, G. Yuan, P. Deng, D. Sengupta, Z. Cheng, Y. Cao, J. Ren, Y. Qin, Y. Zhou, Y. Jia, O. Gozani, D. J. Patel, Z. Wang, Molecular basis of nucleosomal H3K36 methylation by NSD methyltransferases. *Nature* **590**, 498–503 (2021).

26. N. Dephoure, C. Zhou, J. Villén, S. A. Beausoleil, C. E. Bakalarski, S. J. Elledge, S. P. Gygi, A quantitative atlas of mitotic phosphorylation. *Proc. Natl. Acad. Sci. U.S.A.* **105**, 10762–10767 (2008).

27. P. Mertins, F. Yang, T. Liu, D. R. Mani, V. A. Petyuk, M. A. Gillette, K. R. Clauser, J. W. Qiao, M. A. Gritsenko, R. J. Moore, D. A. Levine, R. Townsend, P. Erdmann-Gilmore, J. E. Snider, S. R. Davies, K. V. Ruggles, D. Fenyo, R. T. Kitchens, S. Li, N. Olvera, F. Dao, H. Rodriguez, D. W. Chan, D. Liebler, F. White, K. D. Rodland, G. B. Mills, R. D. Smith, A. G. Paulovich, M. Ellis, S. A. Carr, Ischemia in tumors induces early and sustained phosphorylation changes in stress kinase pathways but does not affect global protein levels. *Mol. Cell. Proteomics* **13**, 1690–1704 (2014).

28. M. Klammer, M. Kaminski, A. Zedler, F. Oppermann, S. Blencke, S. Marx, S. Müller, A. Tebbe, K. Godl, C. Schaab, Phosphosignature predicts dasatinib response in non-small cell lung cancer. *Mol. Cell. Proteomics* **11**, 651–668 (2012).

29. M. Franz-Wachtel, S. A. Eisler, K. Krug, S. Wahl, A. Carpy, A. Nordheim, K. Pfizenmaier, A. Hausser, B. Macek, Global detection of protein kinase D-dependent phosphorylation events in nocodazole-treated human cells. *Mol. Cell. Proteomics* **11**, 160–170 (2012).

30. P. Beli, N. Lukashchuk, S. A. Wagner, B. T. Weinert, J. V. Olsen, L. Baskcomb, M. Mann, S. P. Jackson, C. Choudhary, Proteomic investigations reveal a role for RNA processing factor THRAP3 in the DNA damage response. *Mol. Cell* **46**, 212–225 (2012).

31. C. Weber, T. B. Schreiber, H. Daub, Dual phosphoproteomics and chemical proteomics analysis of erlotinib and gefitinib interference in acute myeloid leukemia cells. *J. Proteomics* **75**, 1343–1356 (2012).

32. Z. Q. Wu, X. Yang, G. Weber, X. Liu, Plk1 phosphorylation of TRF1 is essential for its binding to telomeres. *J. Biol. Chem.* **283**, 25503–25513 (2008).

33. Z. Yang, K. Sharma, T. de Lange, TRF1 uses a noncanonical function of TFIIH to promote telomere replication. *Genes Dev.* **36**, 956–969 (2022).

34. M. Zimmermann, T. Kibe, S. Kabir, T. de Lange, TRF1 negotiates TTAGGG repeat-associated replication problems by recruiting the BLM helicase and the TPP1/POT1 repressor of ATR signaling. *Genes Dev.* **28**, 2477–2491 (2014).

35. A. Sfeir, S. T. Kosiyatrakul, D. Hockemeyer, S. L. MacRae, J. Karlseder, C. L. Schildkraut, T. de Lange, Mammalian telomeres resemble fragile sites and require TRF1 for efficient replication. *Cell* **138**, 90–103 (2009).

36. R. M. Marion, I. L. de Silanes, L. Mosteiro, B. Gamache, M. Abad, C. Guerra, D. Megías, M. Serrano, M. A. Blasco, Common telomere changes during in vivo reprogramming and early stages of tumorigenesis. *Stem Cell Reports* **8**, 460–475 (2017).

37. R. M. Marión, J. J. Montero, I. López de Silanes, O. Graña-Castro, P. Martínez, S. Schoeftner, J. A. Palacios-Fábrega, M. A. Blasco, TERRA regulate the transcriptional landscape of pluripotent cells through TRF1-dependent recruitment of PRC2. *eLife* **8**, e44656 (2019).

38. T. Simonet, L. E. Zaragosi, C. Philippe, K. Lebrigand, C. Schouteden, A. Augereau, S. Bauwens, J. Ye, M. Santagostino, E. Giulotto, F. Magdinier, B. Horard, P. Barbry, R. Waldmann, E. Gilson, The human TTAGGG repeat factors 1 and 2 bind to a subset of interstitial telomeric sequences and satellite repeats. *Cell Res.* **21**, 1028–1038 (2011).

39. Y. Zhou, Y. Wang, K. Krause, T. Yang, J. A. Dongus, Y. Zhang, F. Turck, Telobox motifs recruit CLF/SWN–PRC2 for H3K27me3 deposition via TRB factors in Arabidopsis. *Nat. Genet.* **50**, 638–644 (2018).

40. A. K. Michael, R. S. Grand, L. Isbel, S. Cavadini, Z. Kozicka, G. Kempf, R. D. Bunker, A. D. Schenk, A. Graff-Meyer, G. R. Pathare, J. Weiss, S. Matsumoto, L. Burger, D. Schübeler, N. H. Thomä, Mechanisms of OCT4-SOX2 motif readout on nucleosomes. *Science* **368**, 1460–1465 (2020).

41. J. Déjardin, R. E. Kingston, Purification of proteins associated with specific genomic Loci. *Cell* **136**, 175–186 (2009).

42. H. Huang, B. R. Sabari, B. A. Garcia, C. D. Allis, Y. Zhao, SnapShot: Histone modifications. *Cell* **159**, 458–458.e1 (2014).

43. G. E. Ghanim, A. J. Fountain, A. M. M. van Roon, R. Rangan, R. das, K. Collins, T. H. D. Nguyen, Structure of human telomerase holoenzyme with bound telomeric DNA. *Nature* **593**, 449–453 (2021).

44. Z. Sekne, G. E. Ghanim, A.-M. M. van Roon, T. H. D. Nguyen, Structural basis of human telomerase recruitment by TPP1-POT1. *Science* **375**, 1173–1176 (2022).

45. F. Weissmann, G. Petzold, R. VanderLinden, P. J. Huis in 't Veld, N. G. Brown, F. Lampert, S. Westermann, H. Stark, B. A. Schulman, J. M. Peters, biGBac enables rapid gene assembly for the

expression of large multisubunit protein complexes. *Proc. Natl. Acad. Sci. U.S.A.* **113**, E2564–2569 (2016).

46. M. T. Marty, A. J. Baldwin, E. G. Marklund, G. K. A. Hochberg, J. L. P. Benesch, C. V. Robinson, Bayesian deconvolution of mass and ion mobility spectra: From binary interactions to polydisperse ensembles. *Anal. Chem.* **87**, 4370–4376 (2015).

47. J. Zivanov, J. Otón, Z. Ke, A. von Kügelgen, E. Pyle, K. Qu, D. Morado, D. Castaño-Díez, G. Zanetti, T. A. M. Bharat, J. A. G. Briggs, S. H. W. Scheres, A Bayesian approach to single-particle electron cryo-tomography in RELION-4.0. *eLife* **11**, e83724 (2022).

48. D. Kimanius, L. Dong, G. Sharov, T. Nakane, S. H. W. Scheres, New tools for automated cryo-EM single-particle analysis in RELION-4.0. *Biochem. J.* **478**, 4169–4185 (2021).

49. A. Rohou, N. Grigorieff, CTFFIND4: Fast and accurate defocus estimation from electron micrographs. *J. Struct. Biol.* **192**, 216–221 (2015).

50. I. M. Nodelman, S. das, A. M. Faustino, S. D. Fried, G. D. Bowman, J. P. Armache, Nucleosome recognition and DNA distortion by the Chd1 remodeler in a nucleotide-free state. *Nat. Struct. Mol. Biol.* **29**, 121–129 (2022).

51. J. Zivanov, T. Nakane, B. O. Forsberg, D. Kimanius, W. J. H. Hagen, E. Lindahl, S. H. W. Scheres, New tools for automated high-resolution cryo-EM structure determination in RELION-3. *eLife* **7**, e42166 (2018).

52. T. Bepler, A. Morin, M. Rapp, J. Brasch, L. Shapiro, A. J. Noble, B. Berger, Positive-unlabeled convolutional neural networks for particle picking in cryo-electron micrographs. *Nat. Methods* **16**, 1153–1160 (2019).

53. J. Zivanov, T. Nakane, S. H. W. Scheres, Estimation of high-order aberrations and anisotropic magnification from cryo-EM data sets in *RELION*-3.1. *IUCrJ* **7**, 253–267 (2020).

54. P. B. Rosenthal, R. Henderson, Optimal determination of particle orientation, absolute hand, and contrast loss in single-particle electron cryomicroscopy. *J. Mol. Biol.* **333**, 721–745 (2003).

55. S. Chen, G. McMullan, A. R. Faruqi, G. N. Murshudov, J. M. Short, S. H. W. Scheres, R. Henderson, High-resolution noise substitution to measure overfitting and validate resolution in 3D structure determination by single particle electron cryomicroscopy. *Ultramicroscopy* **135**, 24–35 (2013).

56. Y. Z. Tan, P. R. Baldwin, J. H. Davis, J. R. Williamson, C. S. Potter, B. Carragher, D. Lyumkis, Addressing preferred specimen orientation in single-particle cryo-EM through tilting. *Nat. Methods* **14**, 793–796 (2017).

57. A. Punjani, J. L. Rubinstein, D. J. Fleet, M. A. Brubaker, cryoSPARC: Algorithms for rapid unsupervised cryo-EM structure determination. *Nat. Methods* **14**, 290–296 (2017).

58. A. Punjani, H. Zhang, D. J. Fleet, Non-uniform refinement: Adaptive regularization improves single-particle cryo-EM reconstruction. *Nat. Methods* **17**, 1214–1221 (2020).

59. W. J. H. Hagen, W. Wan, J. A. G. Briggs, Implementation of a cryo-electron tomography tilt-scheme optimized for high resolution subtomogram averaging. *J. Struct. Biol.* **197**, 191–198 (2017).

60. D. N. Mastronarde, S. R. Held, Automated tilt series alignment and tomographic reconstruction in IMOD. *J. Struct. Biol.* **197**, 102–113 (2017).

61. E. F. Pettersen, T. D. Goddard, C. C. Huang, G. S. Couch, D. M. Greenblatt, E. C. Meng, T. E. Ferrin, UCSF Chimera—A visualization system for exploratory research and analysis. *J. Comput. Chem.* **25**, 1605–1612 (2004).

62. A. Casañal, B. Lohkamp, P. Emsley, Current developments in Coot for macromolecular model building of Electron Cryo-microscopy and Crystallographic Data. *Protein Sci.* **29**, 1055–1064 (2020).

63. G. N. Murshudov, P. Skubák, A. A. Lebedev, N. S. Pannu, R. A. Steiner, R. A. Nicholls, M. D. Winn, F. Long, A. A. Vagin, REFMAC5 for the refinement of macromolecular crystal structures. *Acta Crystallogr. D Biol. Crystallogr.* **67**, 355–367 (2011).

64. P. V. Afonine, R. W. Grosse-Kunstleve, N. Echols, J. J. Headd, N. W. Moriarty, M. Mustyakimov, T. C. Terwilliger, A. Urzhumtsev, P. H. Zwart, P. D. Adams, Towards automated crystallographic structure refinement with phenix.refine. *Acta Crystallogr. D Biol. Crystallogr.* **68**, 352–367 (2012).

65. R. A. Nicholls, M. Fischer, S. McNicholas, G. N. Murshudov, Conformation-independent structural comparison of macromolecules with ProSMART. *Acta Crystallogr. D Biol. Crystallogr.* **70**, 2487–2499 (2014).

66. A. Brown, F. Long, R. A. Nicholls, J. Toots, P. Emsley, G. Murshudov, Tools for macromolecular model building and refinement into electron cryo-microscopy reconstructions. *Acta Crystallogr. D Biol. Crystallogr.* **71**, 136–153 (2015).

67. D. Liebschner, P. V. Afonine, M. L. Baker, G. Bunkóczi, V. B. Chen, T. I. Croll, B. Hintze, L. W. Hung, S. Jain, A. J. McCoy, N. W. Moriarty, R. D. Oeffner, B. K. Poon, M. G. Prisant, R. J. Read, J. S. Richardson, D. C. Richardson, M. D. Sammito, O. V. Sobolev, D. H. Stockwell, T. C. Terwilliger, A. G. Urzhumtsev, L. L. Videau, C. J. Williams, P. D. Adams, Macromolecular structure determination using x-rays, neutrons and electrons: Recent developments in Phenix. *Acta Crystallogr. D Biol. Crystallogr.* **75**, 861–877 (2019).

68. C. J. Williams, J. J. Headd, N. W. Moriarty, M. G. Prisant, L. L. Videau, L. N. Deis, V. Verma, D. A. Keedy, B. J. Hintze, V. B. Chen, S. Jain, S. M. Lewis, W. B. Arendall III, J. Snoeyink, P. D. Adams, S. C. Lovell, J. S. Richardson, D. C. Richardson, MolProbity: More and better reference data for improved all-atom structure validation. *Protein Sci.* **27**, 293–315 (2018).

69. T. D. Goddard, C. C. Huang, E. C. Meng, E. F. Pettersen, G. S. Couch, J. H. Morris, T. E. Ferrin, UCSF ChimeraX: Meeting modern challenges in visualization and analysis. *Protein Sci.* **27**, 14–25 (2018).

70. F. Sievers, A. Wilm, D. Dineen, T. J. Gibson, K. Karplus, W. Li, R. Lopez, H. McWilliam, M. Remmert, J. Söding, J. D. Thompson, D. G. Higgins, Fast, scalable generation of high-quality protein multiple sequence alignments using Clustal Omega. *Mol. Syst. Biol.* **7**, 539 (2011).

71. X. Robert, P. Gouet, Deciphering key features in protein structures with the new ENDscript server. *Nucleic Acids Res.* **42**, W320–W324 (2014).

72. A. V. Colasanti, X.-J. Lu, W. K. Olson, Analyzing and building nucleic acid structures with 3DNA. *J. Vis. Exp.*, e4401 (2013).

73. S. Eustermann, K. Schall, D. Kostrewa, K. Lakomek, M. Strauss, M. Moldt, K. P. Hopfner, Structural basis for ATP-dependent chromatin remodelling by the INO80 complex. *Nature* **556**, 386–390 (2018).

74. J. Bednar, I. Garcia-Saez, R. Boopathi, A. R. Cutter, G. Papai, A. Reymer, S. H. Syed, I. N. Lone, O. Tonchev, C. Crucifix, H. Menoni, C. Papin, D. A. Skoufias, H. Kurumizaka, R. Lavery, A. Hamiche, J. J. Hayes, P. Schultz, D. Angelov, C. Petosa, S. Dimitrov, Structure and dynamics of a 197 bp nucleosome in complex with linker histone H1. *Mol. Cell* **66**, 384–397.e8 (2017).
